# Grip strength: are some adiposity phenotypes more detrimental than others? A Mendelian randomization study

**DOI:** 10.1002/oby.24339

**Published:** 2025-07-17

**Authors:** Amy E. Taylor, John Vincent, Dylan M. Williams, Rachel Cooper, Snehal M. Pinto Pereira

**Affiliations:** ^1^ Institute of Sport, Exercise and Health, Division of Surgery and Interventional Science, Faculty of Medical Sciences University College London London UK; ^2^ Division of Psychiatry University College London London UK; ^3^ Unit for Lifelong Health and Ageing University College London London UK; ^4^ AGE Research Group, Translational and Clinical Research Institute, Faculty of Medical Sciences Newcastle University Newcastle upon Tyne UK; ^5^ National Institute for Health and Care Research Newcastle Biomedical Research Centre Newcastle upon Tyne Hospitals NHS Foundation Trust, Cumbria, Northumberland, Tyne and Wear NHS Foundation Trust, Newcastle University Newcastle upon Tyne UK

## Abstract

**Objective:**

Our objective was to investigate causal associations of adiposity in different locations and metabolically favorable and unfavorable adiposity (MetFA and MetUFA, respectively) with grip strength.

**Methods:**

Observational cross‐sectional and Mendelian randomization (MR) (sex combined and stratified) analysis within UK Biobank (*N* ≤ 340,258) was used to assess the relationships of  visceral, abdominal subcutaneous, and gluteofemoral adipose tissue, anterior and posterior thigh muscle fat infiltration (ATMFI and PTMFI, respectively), body fat (BF) percentage, MetFA, and MetUFA with grip strength.

**Results:**

In inverse variance weighted MR analysis, SD increases in BF, MetFA, and ATMFI were associated with lower grip strength by the following: −0.10 SD (95% CI: −0.16 to −0.04), −0.31 SD (95% CI: −0.45 to −0.18), and −0.05 SD (95% CI: −0.09 to −0.01), respectively. PTMFI associations aligned with ATMFI. Observational analyses were consistent for BF and ATMFI/PTMFI, but weighted median/mode MR corroborated findings for MetFA and ATMFI/PTMFI only. Higher visceral adipose tissue was associated with lower grip strength in observational analyses only. Associations for higher abdominal subcutaneous adipose tissue were inconsistent: Observational analyses suggested weaker grip; MR analyses suggested stronger grip, particularly in female individuals. There was no strong evidence in MR for associations with MetUFA or gluteofemoral adipose tissue.

**Conclusions:**

Targeting fat infiltration in muscle may improve muscle function. MetFA appears to negatively impact muscle strength, requiring further investigation into underlying mechanisms.

**Study Importance**

**What is already known?**

- There are strong links between adiposity and muscle strength, but the direction and strength of association may vary over the life course.
- By mid‐ to later life, overall adiposity appears to have a net detrimental effect on grip strength (a commonly used proxy for muscle strength), yet little is understood regarding how location or type (metabolically favorable or unfavorable) of adiposity affects grip strength.

**What does this study add?**

- Using Mendelian randomization, we found that muscle fat infiltration has a negative impact on grip strength, but there was limited evidence for effects of visceral, subcutaneous, or gluteofemoral adipose tissue.
- There was also strong evidence for a detrimental role of so‐called “metabolically favorable” adiposity on grip strength but no consistent association between metabolically unfavorable adiposity and grip strength.

**How might these results change the direction of research or the focus of clinical practice?**

- Future research should focus on developing interventions to prevent accumulation of and/or reduce fat in muscle in order to maintain muscle strength in mid‐ to later life.
- Improved characterization of metabolically favorable adiposity is needed to better understand pathways via which it leads to lower muscle strength.

## INTRODUCTION

Globally, an estimated 11% of people over 60 years old may have sarcopenic obesity, a condition characterized by increased adiposity with reduced muscle mass and function [1]. Given the high prevalence of obesity and increases in body fat (BF) with aging [2], an important research focus is on understanding how adiposity affects muscle function, in particular muscle strength. This knowledge can be used to inform future intervention strategies to prevent sarcopenia.

At least two challenges complicate understanding links between adiposity and muscle strength. First, the relationship may vary over the life course. At younger ages, excess body weight can stimulate muscle growth and strength [2]. However, prolonged exposure to adiposity may reduce muscle strength [2, 3]. Second, the method used to measure adiposity can influence findings. We previously showed that higher body mass index (BMI; which combines fat and lean mass) was associated with greater grip strength in men [4]. In contrast, excess BF percentage (BF%) was detrimental to grip strength [4].

Evidence is growing that the location of adipose tissue influences the adiposity─muscle strength relationship. Studies show higher waist circumference is linked to lower grip strength independent of BMI across wide age ranges (18–64 and 48–98 years) [5, 6]. Similarly, higher waist‐hip ratio is associated with lower grip strength in men (40–70 years) in the UK Biobank (though evidence is less consistent for women) [4]. These observations align with established research indicating that central adiposity, in particular visceral adipose tissue (VAT), is an independent risk factor for obesity‐related conditions, such as coronary heart disease, diabetes, and some cancers [7, 8]. More recently, attention has focused on fat deposition within and across skeletal muscle fibers, known as muscle fat infiltration (MFI) [9]. Although research into MFI is limited due to difficulties measuring and characterizing MFI at scale, studies in older adults (>70 years) show MFI is associated with mobility loss [10] and decreased leg muscle strength and power [11].

Given the heterogeneity in metabolic dysregulation according to adipose tissue location [12], it is important to understand whether observed associations between central adiposity measures and grip strength are driven by differences in metabolic activity of adipose tissue rather than adipose tissue location per se. For example, up to 40% of individuals with obesity do not (yet) have obesity‐related health conditions, suggesting that different types of adiposity, with varying metabolic profiles, may exist [13]. Recently, genetic studies have identified genetic variants associated with “unfavorable adiposity,” here referred to as metabolically unfavorable adiposity (MetUFA), characterized by adiposity that is associated with adverse metabolic profiles, higher levels of ectopic fat, and increased cardiometabolic disease risk. Conversely, “favorable adiposity,” referred to here as metabolically favorable adiposity (MetFA), is adiposity associated with more favorable metabolic profiles and reduced cardiometabolic risk [13]. Although MetFA and MetUFA are genetic concepts that do not exist as measurable phenotypes, their genetic scores can be used to investigate whether pathways between adiposity and health outcomes are likely to be due to adverse metabolic effects of obesity [14].

Current research on adiposity distribution and muscle strength is limited by the use of general measures of adiposity, including waist circumference and waist‐hip ratio, which do not fully capture all potentially meaningful variation in fat tissue distribution [4]. Observational studies may also be affected by confounding and reverse causality. Mendelian randomization (MR), which uses genetic variants as instrumental variables, offers an alternative methodology to understand these relationships [15]. MR, using data from genome‐wide association studies (GWAS) with advanced imaging techniques like magnetic resonance imaging (MRI) [16, 17], can clarify how different adiposity locations (e.g., VAT, MFI) influence muscle strength.

Using individual‐level data from over 340,000 UK Biobank participants, we aimed to investigate 1) the average effects over a lifetime of adiposity in different locations (e.g., VAT, MFI) on grip strength; and 2) whether there are differences in effects of MetFA and MetUFA tissue on grip strength.

## METHODS

We used data from the UK Biobank, a study of over 500,000 adults aged 37–73 years from the United Kingdom, at enrollment in 2006–2010 [18]. All enrolled participants attended a recruitment center where they provided sociodemographic and lifestyle and health‐related information and undertook a range of physical measurements. Participants provided blood and saliva samples, from which genome‐wide single‐nucleotide polymorphism (SNP) data were generated. Quality control for genetic data was performed centrally by the UK Biobank [19]. From 2014 onward, participants have been invited to undergo brain, cardiac, and abdominal MRI, with the aim of scanning 100,000 participants [20]. At imaging visits, participants answered questionnaires and undertook physical measures using the same protocols as the baseline visit. This study includes up to 340,528 unrelated individuals of European ancestry (Figure S1). The UK Biobank received approval from the National Information Governance Board for Health and Social Care and the National Health Service North West Centre for Research Ethics Committee (reference: 11/NW/0382; online Supporting Information Methods 1.1). All participating individuals provided informed consent.

### Adiposity measures

Full details are available elsewhere [16, 21, 22] and in online Supporting Information Methods 1.2. Briefly, BF% was estimated via bioelectrical impedance in participants at baseline (*N* = 491,751) and the imaging visit (*n* = 55,638). Up to 56,000 participants had MRI scans from neck to knees [21]. VAT, abdominal subcutaneous adipose tissue (ASAT), gluteofemoral adipose tissue (GFAT), anterior thigh MFI (ATMFI), and posterior thigh MFI (PTMFI) were quantified from these scans.

### Grip strength

Grip strength was assessed at baseline and at the imaging visit using a Jamar J00105 hydraulic hand dynamometer while participants were seated upright with their forearms on armrests [23]. Participants were asked to squeeze the handle of the dynamometer as strongly as they could for about 3 s. We use the highest grip strength value (> 0) from either hand taken at the imaging visit (for observational analyses) and at baseline for genetic analyses. Individuals with missing grip strength data (<0.5% of the total sample) were excluded from the analysis.

### Covariates (for observational associations)

Potential confounders were identified a priori: sex, age, standing height, deprivation (assessed by the Townsend deprivation score), smoking status (never, former, current), physical activity (two binary variables: <4 vs. 4+ days/week participants undertook >10 min of moderate activity and vigorous activity), and average alcohol intake over the last year (rarely/never, once/month to twice/week, ≥twice/week) [4]. Deprivation was calculated from home addresses at baseline; all other covariates were measured at the MRI scan visit.

### Adiposity genetic instruments (i.e., SNPs and indels)

We used independent SNPs and indels identified from sex‐combined GWAS of individuals from the UK Biobank as genetic instruments for VAT (*n* = 6), ASAT (*n* = 6), GFAT (*n* = 21) [17], ATMFI (*n* = 17), PTMFI (*n* = 23) [16], and BF% (*n* = 287) [14]. Instruments for MetFA (*n* = 35) and MetUFA (*n* = 37) were identified from a univariate GWAS of BF% and a multivariate GWAS of metabolic biomarkers in the UK Biobank [14]. GWAS were restricted to individuals of European ancestry, apart from those for VAT, ASAT, and GFAT, which comprised 87% of individuals of European ancestry [17]. We used variants listed as independent genetic instruments but performed further clumping using the European populations in LDlink [24] to remove SNPs still in linkage disequilibrium (within 10,000 kilobases [kb] of each other and *R* ^2^ < 0.001). See online Supporting Information Methods 1.3 and Tables S1–S8 for the variants used.

### Statistical methods

Analyses were conducted in Stata version 18 (StataCorp LLC) and RStudio version 4.3.2 (R Project for Statistical Computing). Analyses were performed sex combined and then sex stratified because of previous evidence of sex differences in the effect of adiposity on grip strength [4]. We corrected for multiple testing by applying a Bonferroni correction, dividing 0.05 by 8 adiposity exposures (corrected *p* value threshold = 0.00625).

#### Observational associations

Adiposity and grip strength measures were 1) converted to sex‐specific standard deviation [SD] scores; and 2) divided into sex‐specific quintiles. We calculated correlations and investigated cross‐sectional associations between adiposity measures and grip strength using linear regression. Analyses were adjusted for age, sex, height, smoking, alcohol use, physical activity, and deprivation.

#### Genetic associations

We estimated all two‐way genetic correlations among adiposity measures using GWAS summary statistics [14, 16, 17] via linkage disequilibrium score regression (online Supporting Information Methods 1.4) [25]. To assess how genetically determined MetFA and MetUFA relate to the other adiposity phenotypes, we calculated correlations between genome‐wide significant MetFA and MetUFA polygenic risk scores (PRS) and PRS for the other adiposity phenotypes. We also determined the variance explained in the other adiposity phenotypes by MetFA and MetUFA PRS (online Supporting Information Methods 1.4).

SNP‐exposure (G‐adiposity) associations (βs and standard errors [SE]) were obtained from sex‐combined GWAS [13, 16, 17]. All βs from exposure GWAS represented SD values (online Supporting Information Methods 1.5). As MetFA and MetUFA are genetically defined constructs and not observable phenotypes, in both cases, and as in previous work, G‐adiposity associations for BF% were used [14].

**FIGURE 1:**
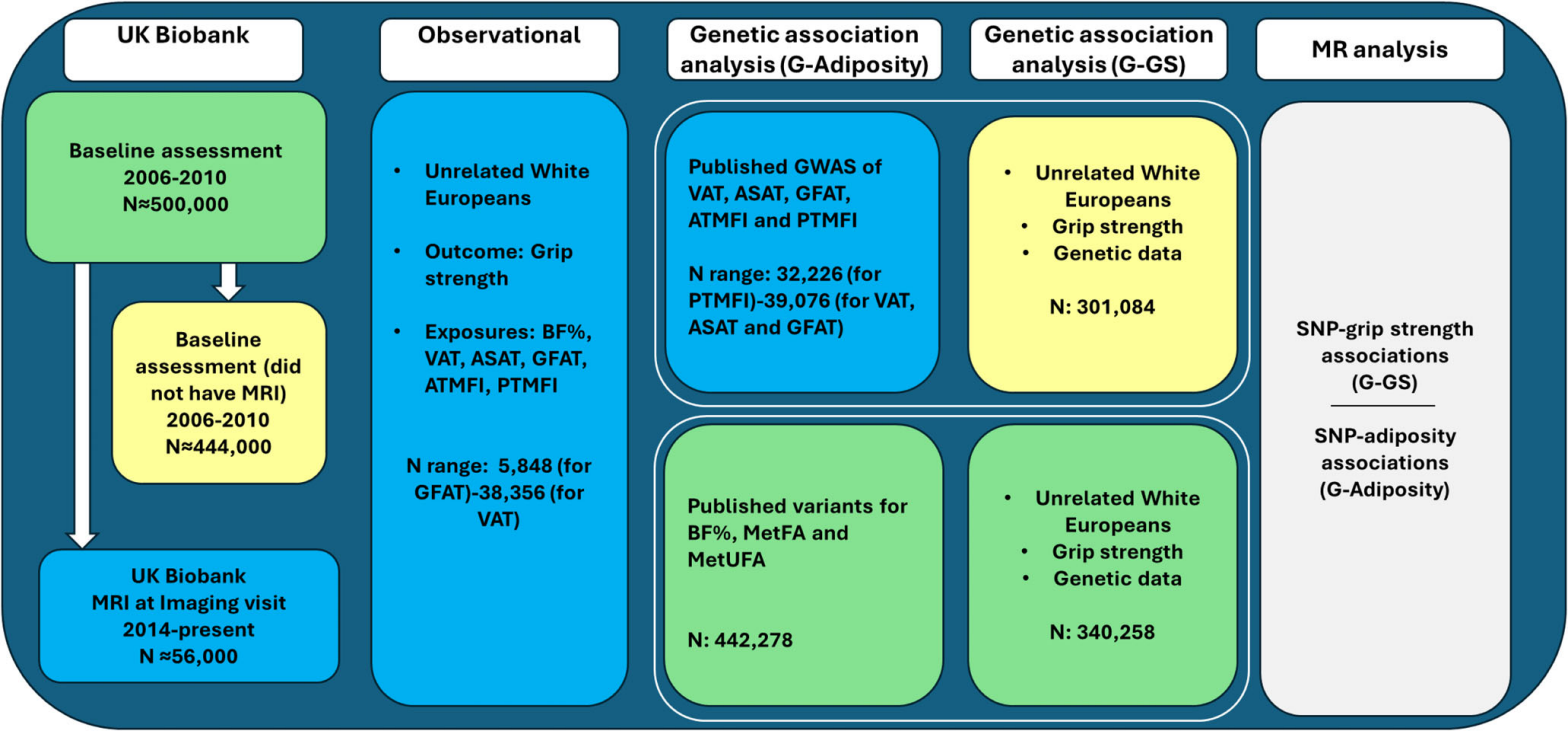
Diagram of the study design for observational and MR analysis. Green: Sample includes all eligible UK Biobank participants. Yellow: Sample includes eligible UK Biobank participants excluding those with MRI data at follow‐up. Blue: Sample includes eligible UK Biobank participants with MRI data. Unrelated White Europeans defined from principal component analysis. Grip strength measured at baseline used in genetic analyses, and grip strength measured at imaging visit used in observational analyses. ASAT, abdominal subcutaneous adipose tissue; ATMFI, anterior thigh muscle fat infiltration; BF, body fat; GFAT, gluteofemoral adipose tissue; G‐GS, G‐grip strength; GWAS, genome‐wide association studies; MetFA, metabolically favorable adiposity; MetUFA, metabolically unfavorable adiposity; MR, Mendelian randomization; PTMFI, posterior thigh muscle fat infiltration; SNP, single‐nucleotide polymorphism; VAT, visceral adipose tissue. [Color figure can be viewed at wileyonlinelibrary.com]

SNP‐outcome (G‐grip strength [G‐GS]) associations were obtained via linear regression of dosage data for each individual SNP on sex‐specific grip strength z scores within the UK Biobank, adjusting for age and 10 genetic principal components. To avoid sample overlap between instrument‐exposure and instrument‐outcome associations for instruments for VAT, ASAT, GFAT, ATMFI, and PTMFI, G‐GS associations were calculated in participants who did not have abdominal MRI data. For BF%, MetFA, and MetUFA, G‐GS associations were calculated in the full sample (Figure 1).

#### MR analysis

MR analyses were performed using the TwoSampleMR package in R (R Project for Statistical Computing) [26]. Associations between adiposity exposures (VAT, ASAT, GFAT, ATMFI, PTMFI, BF%, MetFA, and MetUFA) and grip strength were examined using inverse variance weighted (IVW) random‐effects MR analysis. This method combines the ratios of the individual SNP‐grip strength (G‐GS) and SNP‐adiposity (G‐adiposity) associations using a multiplicative random‐effects meta‐analysis. Each SNP is weighted according to the inverse of the variance of the G‐GS association [27].

To meet underlying assumptions, 1) genetic variants should be associated with the exposure of interest (i.e., adiposity measures); 2) there should be no unmeasured confounders of the instrument‐outcome association (usually due to population stratification); and 3) the instrument should only associate with grip strength via the adiposity measure of interest (i.e., no horizontal pleiotropy) [15]. If underlying assumptions are met, IVW MR is consistent with estimating a causal effect. To test assumption 1, we calculated *F* statistics to estimate the strength of association between each SNP and the exposures: An *F* statistic >10 indicates that weak instrument bias is likely to be small. To test assumption 2, we investigated the extent to which there was evidence for population stratification in the analysis (online Supporting Information Methods 1.8). Heterogeneity for the IVW analysis was assessed via *Q* statistics. A *Q* statistic larger than the degrees of freedom (number of SNPs‐1) indicates the presence of heterogeneity in the analysis and possible bias due to horizontal pleiotropic effects (i.e., violation of assumption 3) [27].

#### Sensitivity analyses

We additionally performed MR analyses using three alternative methods that are more robust to the effects of pleiotropy and rely on different assumptions regarding instrument validity. Specifically, we performed MR weighted median, MR weighted mode, and MR‐Egger. The weighted median method assumes that at least 50% of the weight in the analysis comes from genetic variants that are valid instrumental variables [28]. The weighted mode method assumes that the largest number of similar estimates are derived from valid instruments [29]. For MR‐Egger, assuming pleiotropic effects are independent of the size of associations between the genetic variant and the exposure (the instrument strength independent of direct effect [InSIDE] assumption), the intercept provides an estimate of directional pleiotropy and the slope provides an effect estimate corrected for directional pleiotropy [15].

We also performed radial MR to identify outliers in which there was evidence of heterogeneity in IVW analyses [30], investigated possible bias due to sample overlap, and looked for evidence of selection bias (online Supporting Information Methods 1.6).

## RESULTS

The sample included in our observational analysis (*n* = 38,379) had a mean (SD) age of 66 (7.8) years in male individuals and 64 (7.6) years in female individuals; those included in the genetic analysis (*n* = 301,084) had a mean (SD) age of 57 (8.1) and 57 (7.9) years, respectively (Table 1). Mean (SD) grip strength was 39.2 (8.7) kg and 24.0 (6.1) kg in the observational sample and 41.7 (8.9) kg and 25.0 (6.3) kg in the genetic sample for male and female individuals, respectively. Observational analyses for GFAT were based on a smaller sample (*n* = 6121; details in online Supporting Information Methods 1.2). The characteristics of the full UK Biobank sample (restricted to unrelated Europeans) were similar to the sample excluding participants with MRI data (Table S9).

**TABLE 1:** Characteristics of sub‐samples of UK Biobank participants included in observational and genetic analyses.

	Observational sample^a^ (*n* = 38,379)		Genetic sample excluding participants with abdominal MRI data^b^ (*n* = 301,084)	
	Males	Females	Males	Females
*n*	18,821 (49%)	19,558 (51%)	138,184 (46%)	162,900 (54%)
Outcome				
Grip strength (kg)	39.2 (8.7)	24.0 (6.1)	41.7 (8.9)	25.0 (6.3)
Exposures				
BF (%)	25.7 (5.7)	36.2 (6.7)	25.4 (5.8)	36.7 (6.9)
VAT (L)	5.0 (2.4)	2.7 (1.5)	−	
ASAT (L)	5.8 (2.4)	7.8 (3.3)	−	
GFAT (L)^c^	9.1 (2.5)	11.3 (3.2)	−	
ATMFI (%)	6.9 (1.8)	7.9 (1.9)	−	
PTMFI (%)	10.5 (2.4)	11.7 (2.4)	−	
Covariates				
Age (years)	65.7 (7.8)	64.2 (7.6)	57.3 (8.1)	56.9 (7.9)
Townsend^d^	−2.08 (2.63)	−1.99 (2.64)	−1.47 (3.02)	−1.57 (2.90)
Smoking status				
Never	11,059 (59%)	12,905 (67%)	65,952 (48%)	95,819 (59%)
Former	6,928 (37%)	5,938 (31%)	54,652 (40%)	52,092 (32%)
Current	677 (4%)	509 (3%)	17,044 (12%)	14,398 (9%)
Alcohol intake				
Never/rarely	2,112 (11%)	3,945 (20%)	16,484 (12%)	36,737 (23%)
1/month‐2/week	6,636 (35%)	7,728 (40%)	48,580 (35%)	64,418 (40%)
>2/week	9,971 (53%)	7,760 (40%)	73,013 (53%)	61,625 (38%)
Physical activity				
4+ days/week moderate activity				
Yes	10,300 (56%)	10,953 (58%)	66,877 (50%)	75,134 (49%)
No	8,092 (44%)	7,904 (42%)	65,883 (50%)	77,980 (51%)
4+ days/week vigorous activity				
Yes	4,174 (23%)	3,254 (17%)	29,867 (23%)	23,070 (15%)
No	14,244 (77%)	15,683 (82%)	102,386 (77%)	130,595 (85%)

Note: UK Biobank sample restricted to unrelated individuals of European ancestry with valid grip strength and at least one adiposity measure. Characteristics are *N* (%) or Mean (SD).

Abbreviations: ASAT, abdominal subcutaneous adipose tissue; ATMFI, anterior thigh muscle fat infiltration; BF, body fat; GFAT, gluteofemoral fat; L, litres; PTMFI, posterior thigh muscle fat infiltration; VAT, visceral adipose tissue.

^a^ *n* for exposures/ covariates with missing data (presented for males (M) and females (F) separately): BF M = 17,419, F = 18,139; VAT M = 18,805, F = 19,551; ASAT M = 18,586, F = 19,169; GFAT M = 2,805. F = 3,316; ATMFI M = 17,615, F = 19,365; PTMFI M = 17,621, F = 19,359; Townsend M = 18,808, F = 19,538; Smoking status M = 18,664, F = 19,352; Alcohol M = 18,719, F = 19,433; Moderate activity M = 18,392, F = 18,857; Vigorous activity M = 18,418, F = 18,937.

^b^ *n* for exposures/ covariates with missing data (presented for males (M) and females (F) separately): BF M = 135,415, F = 160,340; Townsend M = 138,012, F = 162,703; Smoking status M = 137,648, F = 162,309; Alcohol M = 138,077, F = 162,780; Moderate activity M = 132,760, F = 153,114; Vigorous activity M = 132,253, F = 153,665.

^c^ Sample with GFAT data had a mean age of 63, SD (7.5) in males and 61, SD (7.3) in females.

^d^ Lower values of Townsend deprivation indicate lower area level deprivation.

### Phenotypic and genetic correlations across adiposity measures

Phenotypic and genetic correlations across adiposity measures were broadly similar (Table 2). For example, BF% was strongly correlated with VAT, ASAT, and GFAT (Pearson correlation coefficients, *r* = 0.7–0.8 [phenotypic], 0.7–0.9 [genetic]) but less so with ATMFI and PTMFI (*r* = 0.5–0.6 [phenotypic and genetic]).

**TABLE 2:** Genetic and phenotypic correlations between regional adiposity variables.

	BF%	VAT	ASAT	GFAT	ATMFI	PTMFI
BF%	1.00	0.71^a^	0.92^a^	0.79^a^	0.55^a^	0.52^a^
VAT	0.76^b^	1.00	0.70^a^	0.49^a^	0.49^a^	0.49^a^
ASAT	0.83^b^	0.72^b^	1.00	0.80^a^	0.49^a^	0.53^a^
GFAT	0.74^b^	0.57^b^	0.78^b^	1.00	0.53^a^	0.57^a^
ATMFI	0.54^b^	0.52^b^	0.45^b^	0.49^b^	1.00	0.93^a^
PTMFI	0.56^b^	0.55^b^	0.48^b^	0.51^b^	0.91^b^	1.00

*Note*: Phenotypic coefficients are Pearson correlation coefficients estimated within the UK Biobank. Correlations are between sex‐specific SD scores. Maximum *n* = 38,859. Genetic correlations are estimated from GWAS data using linkage disequilibrium score regression.

Abbreviations: ASAT, abdominal subcutaneous adipose tissue; ATMFI, anterior thigh muscle fat infiltration; BF, body fat; GFAT, gluteofemoral adipose tissue; PTMFI, posterior thigh muscle fat infiltration; VAT, visceral adipose tissue.

^a^ Genetic correlations.

^b^ Phenotypic correlations.

MetFA and MetUFA PRS were only weakly/moderately correlated with other adiposity PRS (i.e., MetFA from −0.001 for ASAT to 0.33 for GFAT; MetUFA from 0.07 for ATMFI to 0.44 for BF%; Table S10). The variance in measured adiposity explained by MetFA and MetUFA PRS was low (≤1.2%). However, notably, MetFA PRS explained about twice as much variance in measured ATMFI and PTMFI than MetUFA PRS.

### Observational and MR analyses

Linear regressions of sex‐combined and sex‐specific quintiles of all adiposity measures showed that associations between adiposity measures and grip strength were broadly linear (Figures S2–S4). Therefore, we present results per SD increase in adiposity. All instruments had an *F* statistic >10 (Table S11). Harmonized β coefficients, SE values, and results for MR analyses are shown in Tables S12–S28. Although we could not directly test the InSIDE assumption, high correlations among adiposity measures (Table 2) suggest this is unlikely to hold. Therefore, we present results from IVW, weighted median, and weighted mode MR in the main paper; MR‐Egger results are in the online Supporting Information. Given the consistently high correlations between ATMFI and PTMFI (>0.9) and the similarity of estimates, results for ATMFI are presented in the main paper and for PTMFI in the online Supporting Information.

### VAT

**FIGURE 2:**
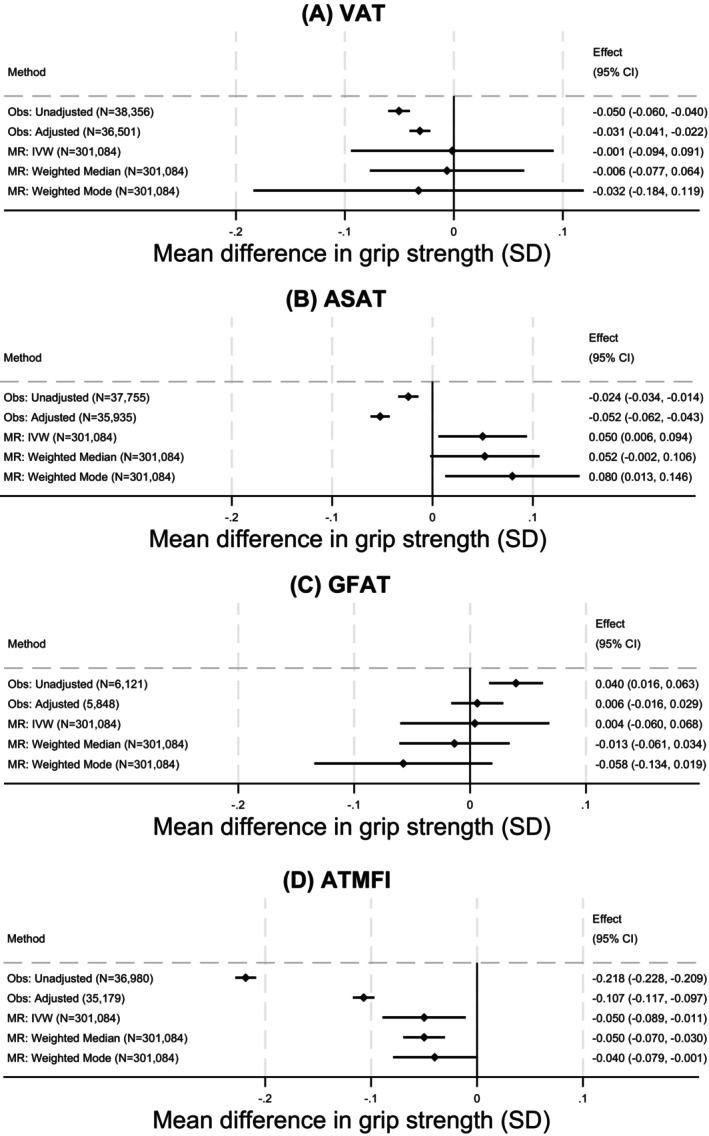
Observed cross‐sectional and MR associations between regional adiposity measures and grip strength. Effect sizes are changes in grip strength per SD increase in adiposity. Observational associations adjusted for age, sex, height, smoking, alcohol use, moderate activity, vigorous activity, and Townsend deprivation. MR analyses adjusted for age, sex, and principal components. ASAT, abdominal subcutaneous adipose tissue; ATMFI, anterior thigh muscle fat infiltration; GFAT, gluteofemoral adipose tissue; IVW, inverse variance weighted; MR, Mendelian randomization; Obs, observational; VAT, visceral adipose tissue.

In observational adjusted analyses, higher VAT was associated with lower grip strength: per 1‐SD higher VAT, grip was lower by 0.03 SD (95% CI: −0.04 to −0.02, *p* = 1.19E‐10; Figure 2A). In MR analyses, the IVW estimate was close to the null but with wide CI values (−0.001 SD [95% CI: −0.09 to 0.09, *p* = 0.98]). There was strong evidence for heterogeneity in the analysis (*p* value from *Q* statistic < 0.001). Findings from median and mode weighted analyses were consistent with the IVW estimate. Results from sex‐stratified analyses were similar (Figure S5 and Table S13).

### ASAT

Observational and MR analyses were inconsistent for ASAT. Adjusted observational analyses showed an inverse association between ASAT and grip strength, such that, per 1‐SD higher ASAT, grip strength was lower by 0.05 SD (95% CI: −0.06 to −0.04, *p* = 5.64E‐27; Figure 2B). However, MR analyses suggested that a 1‐SD higher ASAT was associated with stronger grip (e.g., 0.05 SD [95% CI: 0.01 to 0.09, *p* = 0.03] stronger grip, IVW estimate), although *p* values did not fall below the corrected threshold for significance. There was no clear evidence for heterogeneity (*Q* statistic *p* value = 0.85).

**FIGURE 3:**
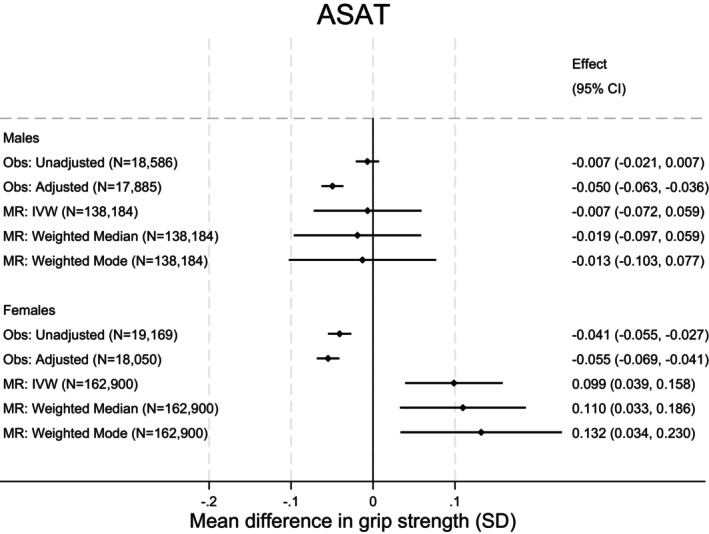
Observed cross‐sectional and MR associations between ASAT and grip strength, stratified by sex. Effect sizes are changes in grip strength per SD increase in ASAT. Observational associations adjusted for age, height, smoking, alcohol use, moderate activity, vigorous activity, and Townsend deprivation. MR analyses adjusted for age, sex, and principal components. ASAT, abdominal subcutaneous adipose tissue; IVW, inverse variance weighted; MR, Mendelian randomization; Obs, observational.

When stratified by sex, observational and MR associations in male individuals were broadly consistent with the null. In female individuals a 1‐SD higher ASAT was associated with a −0.05‐SD lower grip strength (95% CI: −0.06 to −0.03, *p* = 0.007) in observational (adjusted) analysis but a 0.10‐SD higher grip strength (95% CI: 0.04 to 0.16, *p* = 0.001) in MR (IVW) analysis (Figure 3).

### GFAT

There was no clear evidence in adjusted observational or MR analyses for associations between GFAT and grip strength (Figure 2C). Sex‐specific analyses were broadly consistent between sexes (Figure S6).

### MFI

All analyses demonstrated consistent adverse associations between ATMFI and grip strength (Figure 2D). A 1‐SD higher ATMFI was associated with a −0.11‐SD lower grip strength (95% CI: −0.12 to −0.10, *p* < 1E‐16) in observational adjusted analyses and a 0.05‐SD lower grip strength in IVW MR analyses (95% CI: −0.09 to −0.01, *p* = 0.005). IVW MR analysis demonstrated strong evidence for heterogeneity (*Q* statistic *p* value < 0.001), but results from median and mode weighted analyses were highly consistent with the IVW estimate. Results did not vary by sex (Figure S6) and were broadly similar for PTMFI (Figures S8–S9).

### BF%, MetFA, and MetUFA

**FIGURE 4:**
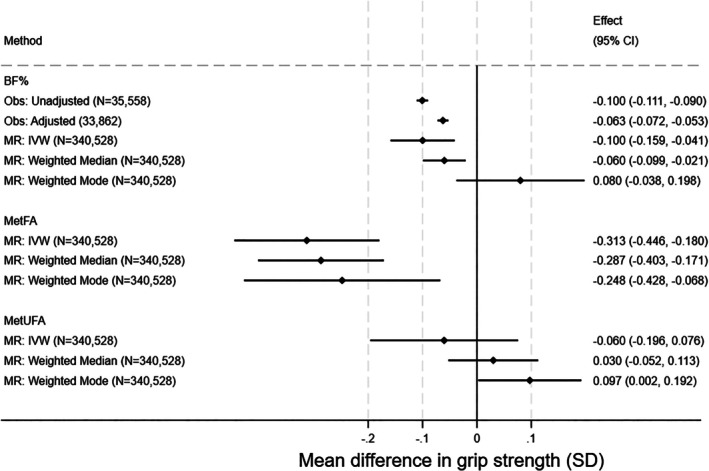
Observed cross‐sectional and MR associations of BF%, MetFA, and MetUFA with and grip strength. Effect sizes for all measures are changes in grip strength per SD increase in BF%. MetFA and MetUFA are not observable entities; therefore, there are no observational analyses for these measures. Observational associations adjusted for age, sex, height, smoking, alcohol use, moderate activity, vigorous activity, and Townsend deprivation. MR analyses adjusted for age, sex, and principal components. BF%, body fat percentage; IVW, inverse variance weighted; MetFA, metabolically favorable adiposity; MetUFA, metabolically unfavorable adiposity; MR, Mendelian randomization; Obs, observational.

Observational and MR associations were consistent for BF%, such that a 1‐SD higher BF% was associated with a 0.06‐SD lower grip strength (95% CI: −0.07 to −0.05, *p* = 9.12E‐36) in observational adjusted analysis and a 0.10‐SD lower grip strength (95% CI: −0.16 to −0.04, *p* = 0.0003) in MR IVW analysis (Figure 4). Sex‐specific analyses were broadly similar (Figure S10).

Higher MetFA was associated with lower grip strength; for example, in MR IVW analysis, a 1‐SD higher BF% was associated with a 0.31‐SD lower grip strength (95% CI: −0.45 to −0.18, *p* = 4.17E‐06). The association between MetUFA and grip strength was smaller and consistent with the null. Sex‐specific MetFA and MetUFA findings were similar to combined sex results (Figure S11).

There was strong evidence for heterogeneity (all *Q* statistic *p* values < 0.001; Tables S23,S25,S27). Results from median weighted MR were consistent with IVW findings, although the effect size was smaller for BF%. Weighted mode analyses results were in the opposite direction to the main analyses for BF% and MetUFA.

### Additional sensitivity analyses

Estimates for sex‐combined MR‐Egger analyses were directionally consistent with MR IVW estimates for all exposures apart from GFAT, BF%, and MetUFA. For BF% there was some evidence of directional pleiotropy (*p* value for intercept = 0.02).

Results from MR IVW analyses did not differ substantially from the main analyses following outlier removal after performing radial MR (Tables S13,S15,S17,S19,S21,S23,S25,S27).

Leave‐one‐out SNP analyses did not materially change any results (Figures S12–S18; Table S28). However, removal of rs56094641 (*FTO*) did attenuate the ASAT results toward the null (Figure S13).

Calculations estimating potential bias due to sample overlap for BF%, MetFA, and MetUFA indicated a low absolute bias (−0.001) and a type 1 error rate of 0.05 (Table S29).

## DISCUSSION

This is the first study to provide a detailed examination of fat types and locations in relation to grip strength using a large, well‐characterized dataset (UK Biobank). By performing both observational and multiple MR analyses (that have different strengths and underlying assumptions) and using multiple indicators of adiposity, we have been able to triangulate findings. As such, our work is uniquely positioned to address the important question of how adiposity affects grip strength in middle‐aged and older adults of European ancestry. Our findings suggest that higher levels of thigh MFI and adiposity that has been classed as “metabolically favorable” are associated with weaker grip strength in both men and women. However, the impact of central adiposity was less clear.

In observational MR IWV and median weighted MR analysis, higher overall adiposity, as assessed by BF%, was adversely associated with grip strength in middle‐ to older‐aged adults. Some caution in interpreting these findings is required due to heterogeneity in MR analyses and opposing directions of results from weighted mode analysis. If our main BF% findings are causal, they suggest that, by middle to late adulthood, higher overall adiposity across the life course has a net detrimental effect on grip strength. These results do not necessarily contradict studies indicating that higher adiposity earlier in life may be associated with stronger grip [31], given that the relationship between adiposity and grip strength varies with age [4]. Furthermore, MR analyses estimate the effect of lifelong exposure to higher adiposity on grip strength at the time of grip strength measurement. This differs from observational analyses that examine a “snapshot” of adiposity at a particular life stage [15].

Our results provide consistent support for MFI causing lower grip strength and, thus, a potential mechanism through which greater overall adiposity leads to lower muscle strength. A 2% absolute increase in ATMFI was associated with a decrease of ∼0.5 kg in grip strength in male individuals and ∼0.3 kg in female individuals. We were limited to exploring thigh MFI. Although these muscles are not engaged when eliciting force during grip strength assessments, it can be assumed that MFI in thigh muscles may be indicative of MFI in other body regions, including upper limbs, in part because of a shared genetic basis. Therefore, efforts should focus on 1) improved methods to measure MFI at scale, which is currently a major challenge [32]; and 2) interventions aimed at preventing and reducing MFI, including physical activity, nutrition, and pharmaceutical intervention. Trials show that physical activity can reduce or prevent MFI accumulation, but more research is needed to determine the most effective duration, type, and intensity [33]. Another important area of future study is the impact of weight loss drugs, such as glucagon‐like peptide‐1 (GLP‐1) receptor agonists. This is because this class of drugs is known to reduce muscle mass [34], but the simultaneous effects on MFI are unknown.

Our results do not provide consistent support for effects of central adiposity or GFAT in relation to grip strength. However, our ability to make strong inferences on VAT and GFAT was limited by wide CI values in our MR analyses. Nevertheless, these findings are supported by the lack of consistent evidence we found for a role of MetUFA, which has been shown previously to be associated with higher VAT and liver and pancreatic fat [13]. Our analysis provides strong evidence that MetFA negatively impacts grip strength. One possible interpretation is that MetFA tends to accumulate in areas where it has a more pronounced effect on muscle strength. This is supported by our finding that PRS for MetFA explained nearly twice the variance in ATMFI and PTMFI compared with MetUFA. Interestingly, higher levels of MetFA have also been associated with lower bone mineral density [35], suggesting that the terms “favorable” and “unfavorable” may need to be reconsidered, especially in relation to musculoskeletal health.

In MR analyses in female individuals, higher ASAT was associated with higher grip strength. This contrasts with observational findings in which higher ASAT was linked to lower grip strength. However, caution is warranted in interpreting these MR results, as the *p* value for the primary MR analysis exceeded the corrected threshold. Additionally, removing the *FTO* SNP, the minor allele of which is associated with both higher lean mass [36] and higher ASAT [17], affected results, suggesting potential pleiotropy. Residual confounding or life‐stage‐specific effects, such as hormonal changes, could also explain the differences between MR and observational results in female individuals. Additional studies are needed to confirm these results and better understand the underlying mechanisms.

We acknowledge study limitations. Although handgrip strength is a commonly used metric for assessing muscle strength, evidence on whether it is an adequate proxy for overall muscle strength is equivocal [37]. Our analysis focused on continuous grip strength, and therefore, findings may not be applicable to low grip strength specifically. This warrants further investigation in future studies using available GWAS data [38]. We intended to investigate effects of regional adiposity measures independent from total adiposity (i.e., BF%) via multivariable MR. However, this was not feasible because of weak instruments (conditional *F* < 10; see online Supporting Information Methods 1.7; Table S30). Given the high phenotypic and genetic correlations observed among adiposity measures, disentangling the effects of these independently on grip strength is complex. One possible method that could be applied is Causal Analysis Using Summary Effect estimates (CAUSE) [39], which uses GWAS summary statistics to account for correlated and uncorrelated pleiotropy. However, adopting this approach is not currently possible as available genome‐wide statistics for grip strength are scaled for weight [40] and are, thus, inappropriate. For the same reason, we did not perform a bidirectional MR analysis to investigate the impact of grip strength on adiposity measures, as interpretation would be problematic due to the adjustment of grip strength GWAS for weight. We propose this as an important next step when more appropriate grip strength instruments are available. We observed evidence for heterogeneity in most MR analyses, and sensitivity analyses for BF and MetUFA were inconsistent with MR IVW. Therefore, these results should be interpreted with caution. We acknowledge that sex‐specific pathways between adiposity and grip strength might exist that we have not identified. Although sex‐specific variants are available for VAT, ASAT, and GFAT [17], they are few in number. Thus, using sex‐specific variants would make results difficult to triangulate as pleiotropy robust methods, such as median weighted and MR‐Egger, could not be used. Our MR analysis assumes associations are linear; reassuringly, most observational associations appeared linear. Our finding of associations between some genetic scores and place of birth suggests a degree of population stratification and/or selection bias (online Supporting Information Methods 1.8 and Figures S19–S21). Replication of our analysis in other populations is therefore advised. Finally, our analysis was restricted to individuals of European ancestry so may not be applicable to other populations.

## CONCLUSION

Our findings strengthen evidence regarding the detrimental role of MFI for grip strength and emphasize the importance of determining the most appropriate MFI treatment and prevention strategies. Identifying when in the life course MFI starts to affect muscle strength, thereby elucidating an appropriate window for intervention, is an important next step. Pathways via which MetFA is linked to lower muscle strength require further investigation, both in terms of identifying potential mediators and characterizing where MetFA is located.

## FUNDING INFORMATION

The work was supported by a UK Medical Research Council senior nonclinical fellowship (reference: MR/Y009398/1) awarded to Snehal M. Pinto Pereira and by funding to support the Unit for Lifelong Health and Ageing at University College London (MC_UU_12019/07). Rachel Cooper acknowledges support from the National Institute for Health and Care Research Newcastle Biomedical Research Centre (reference: NIHR203309). Rachel Cooper also receives support as part of a generous donation made by the McArdle family to Newcastle University for research that will benefit the lives of older people in the UK. The views expressed in the publication are those of the authors and not necessarily those of the funders. The funders had no input into study design; data collection, analysis, and interpretation; the writing of the report; and the decision to submit the article for publication. Researchers were independent of influence from study funders.

## CONFLICT OF INTEREST STATEMENT

The authors declared no conflicts of interest.

## Supporting information


**Data S1.** Supporting Information.

**Data S2.** Tables.

## Data Availability

This research has been conducted using the UK Biobank Resource under application number 71702. Researchers can apply to the UK Biobank to access data. Analysis code for the work conducted in this manuscript is available at https://github.com/amyetaylor/MR_regional_adiposity_grip_strength.
