# Supplementary material for: Grip strength: are some adiposity phenotypes more detrimental than others? A Mendelian randomization study
Source: Obesity (Silver Spring). 2025 Jul 17;33(9):1779–90. doi: 10.1002/oby.24339 (PMC12381625; doi:10.1002/oby.24339)
Supplement: Supplementary file 1 — Data S1. Supporting Information. [file OBY-33-1779-s002.docx]

**Supplementary Methods, Results and Figures**

**Grip strength: are some adiposity phenotypes more detrimental than others? A Mendelian Randomisation study**

Amy E. Taylor^1^, John Vincent^1^, Dylan M. Williams^2, 3^, Rachel Cooper^4,5^, Snehal M Pinto Pereira^1^

1. Institute of Sport, Exercise and Health, Division of Surgery and Interventional Science, Faculty of Medical Sciences, UCL, London, UK

2. Division of Psychiatry, UCL, London, UK

3. Unit for Lifelong Health & Ageing at UCL, London, UK

4. AGE Research Group, Translational and Clinical Research Institute, Faculty of Medical Sciences, Newcastle University, Newcastle upon Tyne, UK

5. NIHR Newcastle Biomedical Research Centre, Newcastle upon Tyne Hospitals NHS Foundation Trust, Cumbria, Northumberland, Tyne and Wear NHS Foundation Trust and Newcastle University, Newcastle upon Tyne, UK

Contents

[1 Supplementary methods 4](#_Toc194915847)

[1.1 Ethical guidelines statement 4](#_Toc194915848)

[1.2 Adiposity measures 4](#_Toc194915849)

[1.3 LD clumping of genetic instruments and gene annotations 4](#_Toc194915850)

[1.4 Calculation of polygenic risk scores and genetic correlations 5](#_Toc194915851)

[1.5 Units of adiposity measures from GWAS 5](#_Toc194915852)

[1.6 Mendelian Randomisation Sensitivity: Additional sensitivity analyses 5](#_Toc194915853)

[1.7 Multivariable MR (MVMR) analysis 5](#_Toc194915854)

[1.8 Investigating selection bias and population stratification in our genetic instruments: methods and results 6](#_Toc194915855)

[2. Supplementary Figures 7](#_Toc194915856)

[Figure S1. Flowchart of the UK Biobank study population 7](#_Toc194915857)

[Figure S2. Unadjusted associations between quintiles of adiposity measures and grip strength, sex-combined, males and females 8](#_Toc194915858)

[Figure S3. Adjusted associations between quintiles of adiposity measures and grip strength, sex-combined, males and females 9](#_Toc194915859)

[Figure S4. Observational associations of PTMFI (Quintiles) with grip strength, sex-combined, males and females 10](#_Toc194915860)

[Figure S5. Sex stratified cross-sectional observational and Mendelian Randomisation analyses of VAT with grip strength 11](#_Toc194915861)

[Figure S6. Sex stratified cross-sectional observational and Mendelian Randomisation analyses of GFAT with grip strength 12](#_Toc194915862)

[Figure S7. Sex stratified cross-sectional observational and Mendelian Randomisation analyses of ATMFI with grip strength 13](#_Toc194915863)

[Figure S8. Cross sectional observational and Mendelian Randomisation analyses of PTMFI with grip strength 14](#_Toc194915864)

[Figure S9. Sex-stratified cross sectional observational and Mendelian Randomisation analyses of PTMFI with grip strength 15](#_Toc194915865)

[Figure S10. Sex stratified cross sectional observational and Mendelian Randomisation analyses of Body fat with grip strength 16](#_Toc194915866)

[Figure S11. Sex stratified Mendelian Randomisation analyses of MetFA and MetUFA with grip strength 17](#_Toc194915867)

[Figure S12. Leave one SNP out analyses for MR of VAT on Grip Strength 18](#_Toc194915868)

[Figure S13. Leave one SNP out analyses for MR of ASAT on Grip Strength 19](#_Toc194915869)

[Figure S14. Leave one SNP out analyses for MR of GFAT on Grip Strength 20](#_Toc194915870)

[Figure S15. Leave one SNP out analyses for MR of ATMFI on Grip Strength 21](#_Toc194915871)

[Figure S16. Leave one SNP out analyses for MR of PTMFI on Grip Strength 22](#_Toc194915872)

[Figure S17. Leave one SNP out analyses for MR of metabolically favourable adiposity on Grip Strength 23](#_Toc194915873)

[Figure S18. Leave one SNP out analyses for MR of metabolically unfavourable adiposity on Grip Strength 24](#_Toc194915874)

[Figure S19. Associations between adiposity measures and genetic scores with sex 25](#_Toc194915875)

[Figure S20. Associations between adiposity measures and genetic scores with place of birth, North coordinate 26](#_Toc194915876)

[Figure S21. Associations between adiposity measures and genetic scores with place of birth, East coordinate 27](#_Toc194915877)

[3. Supplementary References 28](#_Toc194915878)

# 1 Supplementary methods

## 1.1 Ethical guidelines statement

UKB received approval from the National Information Governance Board for Health and Social Care and the National Health Service North West Centre for Research Ethics Committee (Ref: 11/NW/0382). The work has been performed in accordance with the ethical standards laid down in the 1964 Declaration of Helsinki and its later amendments. All persons gave their informed consent to participate in the UK Biobank Study.

## 1.2 Adiposity measures

Body fat percentage (BF%) was estimated via bioelectrical impedance analysis (BIA) using a Tanita BC-418 MA body composition analyser in participants at baseline (N=491,751) and the imaging visit (N= 55,638). Up to 56,000 participants had MRI scans from neck to knees using a Siemens Aera 1.5 T scanner (Syngo MR D13) (Siemens, Erlangen, Germany) (1). Visceral adipose tissue (VAT) and abdominal subcutaneous adipose tissue (ASAT) were quantified from the top of vertebrae T9 to the top of the femoral head. Gluteofemoral adipose tissue (GFAT) was derived by taking the VAT and ASAT volume away from the total adipose tissue between the top of vertebrae T9 and the bottom of the thigh muscles (2). For our analyses, total adipose tissue volume (and therefore GFAT) was only available for a subset of participants (N=8,520) from the UK Biobank data release. Two MFI measures were available: anterior and posterior. Anterior MFI (ATMFI) was calculated as the fat fraction (%) in the quadriceps femoris, sartorius, and tensor fascia latae muscles; posterior MFI (PTMFI) was calculated as the fat fraction (%) in the viable muscle tissue of the gluteus, iliacus, adductor and hamstring muscles (3). For both MFI measures, we took the mean fat fraction from both legs or used the value from one leg when values from the second leg were unavailable. For all MRI measures, we excluded scans with recorded errors.

## 1.3 LD clumping of genetic instruments and gene annotations

We performed LD clumping of reported genomewide significant SNPs and indels using Europeans from the 1000 genomes reference panel (4). We used an R-squared threshold of 0.001 and a clumping window of 10,000kb. This resulted in the following reductions in the number of SNPs used for MR analyses: VAT: 7 SNPs reduced to 6, ASAT: all 6 SNPs included, GFAT: 27 SNPs reduced to 21, ATMFI: all 17 SNPs included, PTMFI: 24 SNPs reduced to 23 SNPs, Body fat percentage: 696 SNPs reduced to 289, MetUFA: 38 SNPs reduced to 37, MetFA: 36 SNPs reduced to 35.

Information on the nearest genes to each SNP for Body fat percentage, MetUFA and MetFA was extracted from the web-based annotation tool SNPNexus (5).

## 1.4 Calculation of polygenic risk scores and genetic correlations

To assess correlations between different fat locations and types, we estimated all two-way genetic correlations between adiposity measures using GWAS summary statistics (3, 6, 7). This was performed via LD score regression using the LDSC tool and LD scores from the 1000 genomes European reference panel (8). Given that full GWAS summary statistics were not available for the BF percentage variants used in the main analyses (9), we used another BF percentage GWAS in Europeans from UK Biobank (10) to estimate genetic correlations.

To enable us to assess how genetically determined MetFA and MetUFA relate to the other adiposity phenotypes, we calculated weighted genome-wide significant polygenic risk scores (PRS) for each adiposity phenotype within UK Biobank by adding together dosage scores for each SNP multiplied by weights from the relevant exposure GWAS. We then calculated correlations between genome wide significant MetFA and MetUFA PRS and PRS for the other adiposity phenotypes and the variance explained in other adiposity phenotypes by MetFA and MetUFA PRS.

## 1.5 Units of adiposity measures from GWAS

VAT, ASAT, GFAT, ATMFI and PTMFI were transformed using rank inverse normal transformation (3, 7). Given that these measures are approximately normally distributed, beta coefficients were assumed to equate to standard deviations (SD). Beta coefficients from the body fat percentage GWAS were in SD units.

## 1.6 Mendelian Randomisation Sensitivity: Additional sensitivity analyses

We performed two further sensitivity analyses where there was evidence of heterogeneity in IVW analysis. Firstly, we used the MR Radial package in R to identify outlying SNPs in all analyses where Q-statistics provided evidence of pleiotropy (11). Outliers are determined with respect to their contribution to global heterogeneity and for each exposure, we used a p-value cut off of 0.05/N SNPs to detect outliers. We then performed MR IVW analyses excluding these SNPs. Secondly, we conducted leave one out analysis to assess if any results were affected by a single genetic variant.

Given that MR analyses for body fat percentage, MetUFA and MetFA have a high degree of sample overlap (using G-Adiposity and G-GS associations from the full UK Biobank sample), we calculated the extent of possible bias due to sample overlap using an online calculator <https://sb452.shinyapps.io/overlap/> (12).

## 1.7 Multivariable MR (MVMR) analysis

Multivariable Mendelian Randomisation is an approach which allows investigation of the direct effect of multiple exposures on an outcome, simultaneously, using genetic instrumental variables (13). To investigate if effects of VAT, ASAT, GFAT, ATMFI and PTMFI were independent of overall bodyfat, we tested the strength of instruments for each phenotype conditional on the body fat percentage SNPs by calculating conditional F-statistics using the MVMR package in R (13). Prior to this we performed LD clumping on the combined set of variants for body fat percentage and each of the regional adiposity variants, in turn, based on Europeans from the 1000 genomes reference panel (4). We used an R-squared threshold of 0.001 and a clumping window of 10,000kb. As recommended, we only performed MVMR analysis when conditional F-statistics for both body fat percentage and the regional adiposity measure were >10 (13). Conditional F-statistics for the regional adiposity measures were all <10 (Supplementary Table S30) so no MVMR analyses were performed.

## 1.8 Investigating selection bias and population stratification in our genetic instruments: methods and results

To investigate whether Mendelian Randomisation analyses were likely to be subject to bias from confounding (population stratification) or selection bias (due to selective participation in UK Biobank), we investigated the association of adiposity genomewide polygenic risk scores (PRS) with variables in UKB that are determined at birth and are not expected to be affected by adiposity (14), specifically: place of birth and sex. At baseline assessment, participants who were born in England, Scotland or Wales were asked which town or district they first lived in and corresponding North and East co-ordinates were recorded (15).

For each adiposity phenotype, PRS were scaled so that a 1 unit increase represented a 1 SD increase in the adiposity measure. Each risk score was then regressed sequentially on sex, north and east coordinates, adjusted for the top 10 genetic principal components within the full genetic sample, but excluding those with MRI data. For sex, regression coefficients represent the risk difference for being male. For comparison, each adiposity phenotype was also regressed on each outcome (16). It should be noted that the phenotypic analysis could only be done within the MRI imaging sample, so phenotypic samples are smaller and are taken at a later timepoint than for the genetic analysis.

The VAT PRS was associated with sex, but biases for all genetic scores were generally much smaller than for corresponding phenotypic measures (Supplementary Figure S19). All PRS were associated with northerly co-ordinates of birthplace to a similar or greater magnitude as the phenotypic measures (Supplementary Figure S20). There was evidence that the BF genetic score was associated with East coordinate (Supplementary Figure S21), but genetic score effect sizes were smaller than those seen for phenotypic measures for VAT, ASAT, GFAT, ATMFI and PTMFI.

Although effect sizes are small, these results suggest that there may be residual confounding by population stratification, with principal components not fully capturing population structure within the UK. In addition, place of birth influences likelihood of being in the UK Biobank sample, so these associations could also be due to selection bias (14).

# 2. Supplementary Figures

## Figure S1. Flowchart of the UK Biobank study population

**
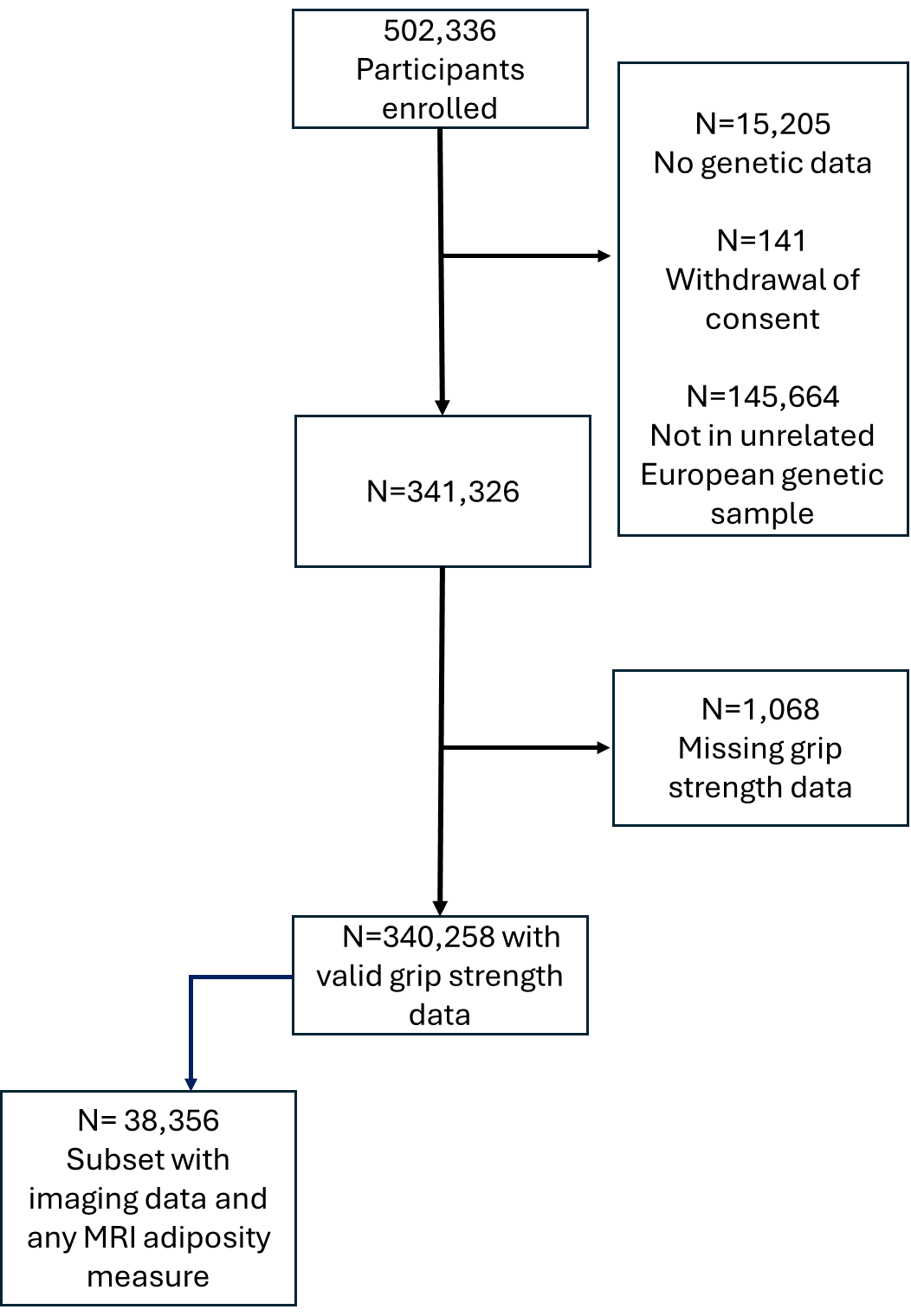
**

## Figure S2. Unadjusted associations between quintiles of adiposity measures and grip strength, sex-combined, males and females

BF: Body fat, VAT: Visceral Adipose Tissue, ASAT: Abdominal Subcutaneous Adipose Tissue, GFAT: Gluteofemoral fat, ATMFI: Anterior Thigh Muscle Fat Infiltration. Q1= lowest quintile, Q5=highest Quintile

## Figure S3. Adjusted associations between quintiles of adiposity measures and grip strength, sex-combined, males and females

BF: Body fat, VAT: Visceral Adipose Tissue, ASAT: Abdominal Subcutaneous Adipose Tissue, GFAT: Gluteofemoral fat, ATMFI: Anterior Thigh Muscle Fat Infiltration. Q1= lowest quintile, Q5=highest Quintile. Associations adjusted for age, sex, smoking, alcohol use, moderate activity, vigorous activity, Townsend deprivation.

## Figure S4. Observational associations of PTMFI (Quintiles) with grip strength, sex-combined, males and females

PTMFI: Posterior Thigh Muscle Fat Infiltration. Q1= lowest quintile, Q5=highest Quintile. Associations adjusted for age, sex, smoking, alcohol use, moderate activity, vigorous activity, Townsend deprivation.

## Figure S5. Sex stratified cross-sectional observational and Mendelian Randomisation analyses of VAT with grip strength

Effect sizes are changes in grip strength per SD increase in adiposity measure. VAT: Visceral Adipose Tissue. Obs: Observational, MR: IVW: Inverse variance weighted Mendelian Randomisation, MR:Weighted Median: Weighted median Mendelian Randomisation, MR Weighted Mode: Weighted Mode Mendelian Randomisation. Observational associations adjusted for age, sex, height, smoking, alcohol use, moderate activity, vigorous activity, Townsend deprivation. MR analyses adjusted for age and principal components

## Figure S6. Sex stratified cross-sectional observational and Mendelian Randomisation analyses of GFAT with grip strength

Effect sizes are changes in grip strength per SD increase in adiposity measure. GFAT: Gluteofemoral adipose tissue. Obs: Observational, MR: IVW: Inverse variance weighted Mendelian Randomisation, MR:Weighted Median: Weighted median Mendelian Randomisation, MR Weighted Mode: Weighted Mode Mendelian Randomisation. Observational associations adjusted for age, sex, height, smoking, alcohol use, moderate activity, vigorous activity, Townsend deprivation. MR analyses adjusted for age and principal components

## Figure S7. Sex stratified cross-sectional observational and Mendelian Randomisation analyses of ATMFI with grip strength

Effect sizes are changes in grip strength per SD increase in adiposity measure. ATMFI: Anterior Thigh Muscle Fat Infiltration. Obs: Observational, MR: IVW: Inverse variance weighted Mendelian Randomisation, MR:Weighted Median: Weighted median Mendelian Randomisation, MR Weighted Mode: Weighted Mode Mendelian Randomisation. Observational associations adjusted for age, sex, height, smoking, alcohol use, moderate activity, vigorous activity, Townsend deprivation. MR analyses adjusted for age and principal components.

## Figure S8. Cross sectional observational and Mendelian Randomisation analyses of PTMFI with grip strength

Effect sizes are changes in grip strength per SD increase in adiposity measure. PTMFI: Posterior Thigh Muscle Fat Infiltration. Obs: Observational, MR: IVW: Inverse variance weighted Mendelian Randomisation, MR:Weighted Median: Weighted median Mendelian Randomisation, MR Weighted Mode: Weighted Mode Mendelian Randomisation. Observational associations adjusted for age, sex, height, smoking, alcohol use, moderate activity, vigorous activity, Townsend deprivation. MR analyses adjusted for age and principal components.

## Figure S9. Sex-stratified cross sectional observational and Mendelian Randomisation analyses of PTMFI with grip strength

Effect sizes are changes in grip strength per SD increase in adiposity measure. PTMFI: Posterior Thigh Muscle Fat Infiltration. Obs: Observational, MR: IVW: Inverse variance weighted Mendelian Randomisation, MR:Weighted Median: Weighted median Mendelian Randomisation, MR Weighted Mode: Weighted Mode Mendelian Randomisation. Observational associations adjusted for age, height, smoking, alcohol use, moderate activity, vigorous activity, Townsend deprivation. MR analyses adjusted for age and principal components.

## Figure S10. Sex stratified cross sectional observational and Mendelian Randomisation analyses of Body fat with grip strength

Effect sizes are changes in grip strength per SD increase in adiposity measure. BF: Bodyfat percentage. Obs: Observational, MR: IVW: Inverse variance weighted Mendelian Randomisation, MR:Weighted Median: Weighted median Mendelian Randomisation, MR Weighted Mode: Weighted Mode Mendelian Randomisation. Observational associations adjusted for age, height, smoking, alcohol use, moderate activity, vigorous activity, Townsend deprivation. MR analyses adjusted for age and principal components.

## Figure S11. Sex stratified Mendelian Randomisation analyses of MetFA and MetUFA with grip strength

Effect sizes are changes in grip strength per SD increase in adiposity measure. MetFA: Metabolically Favourable Adiposity, MetUFA: Metabolically Unfavourable Adiposity. Obs: Observational, MR: IVW: Inverse variance weighted Mendelian Randomisation, MR:Weighted Median: Weighted median Mendelian Randomisation, MR Weighted Mode: Weighted Mode Mendelian Randomisation. Observational associations adjusted for age, height, smoking, alcohol use, moderate activity, vigorous activity, Townsend deprivation. MR analyses adjusted for age and principal components.

## Figure S12. Leave one SNP out analyses for MR of VAT on Grip Strength


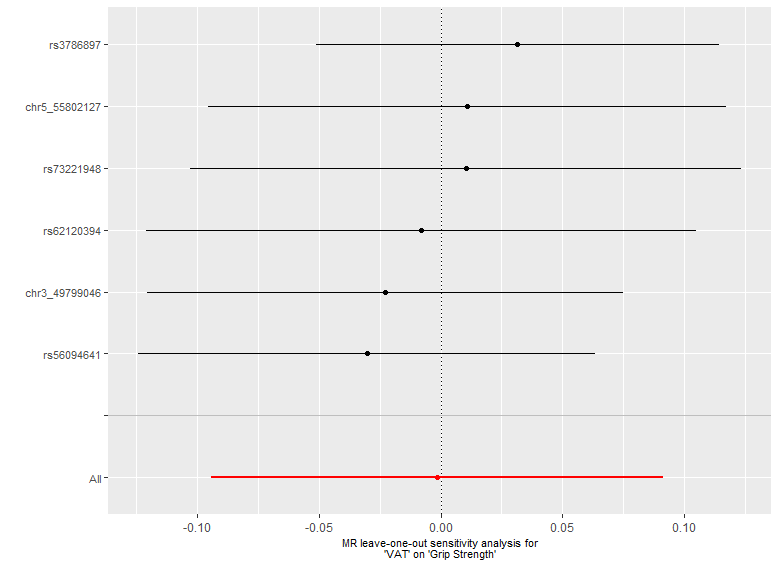


Both


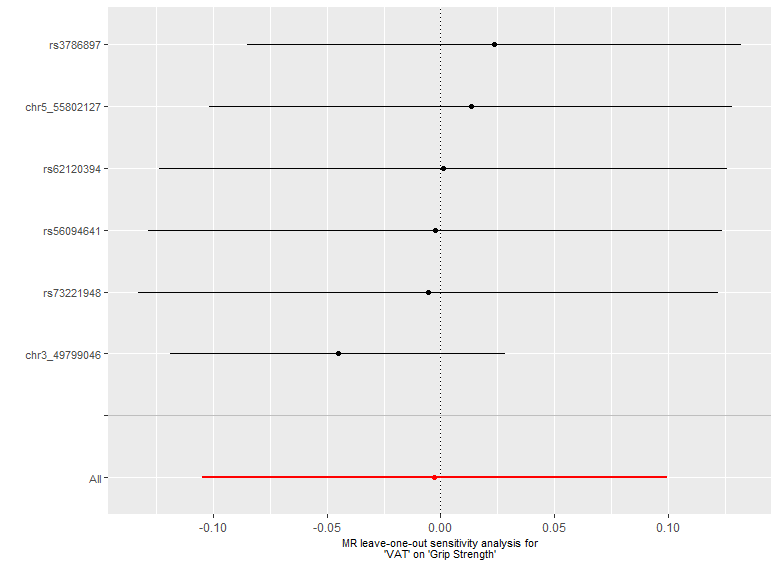
Males


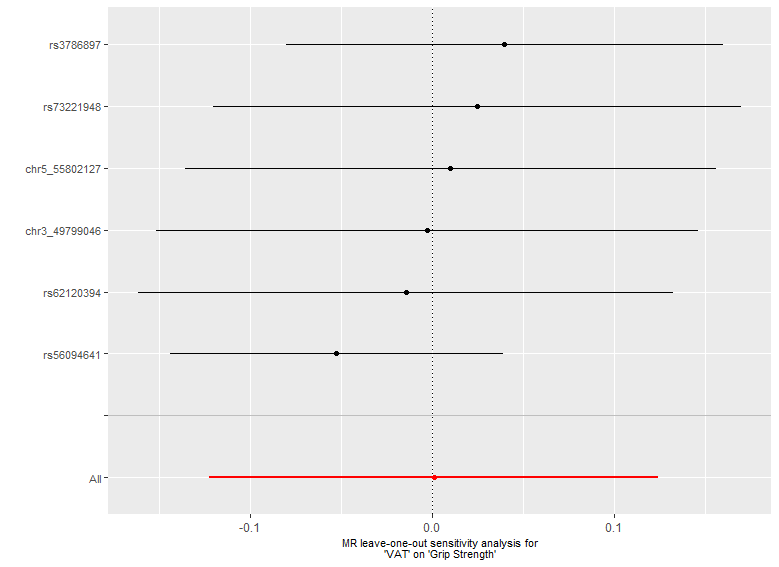
Females

## Figure S13. Leave one SNP out analyses for MR of ASAT on Grip Strength


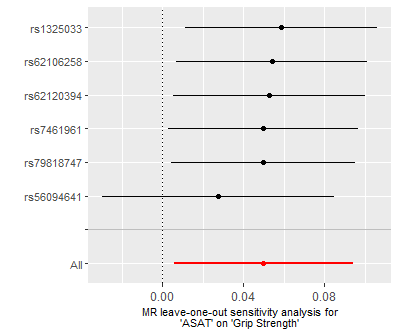


Both


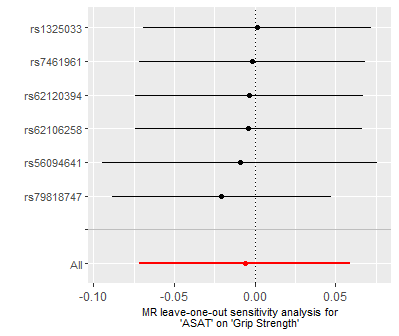
Males


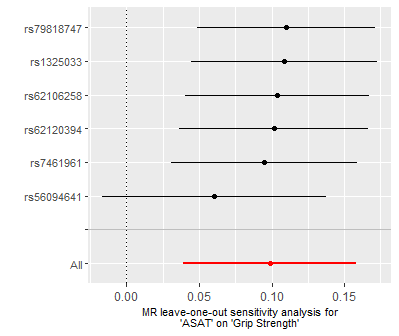
Females

## Figure S14. Leave one SNP out analyses for MR of GFAT on Grip Strength

Both
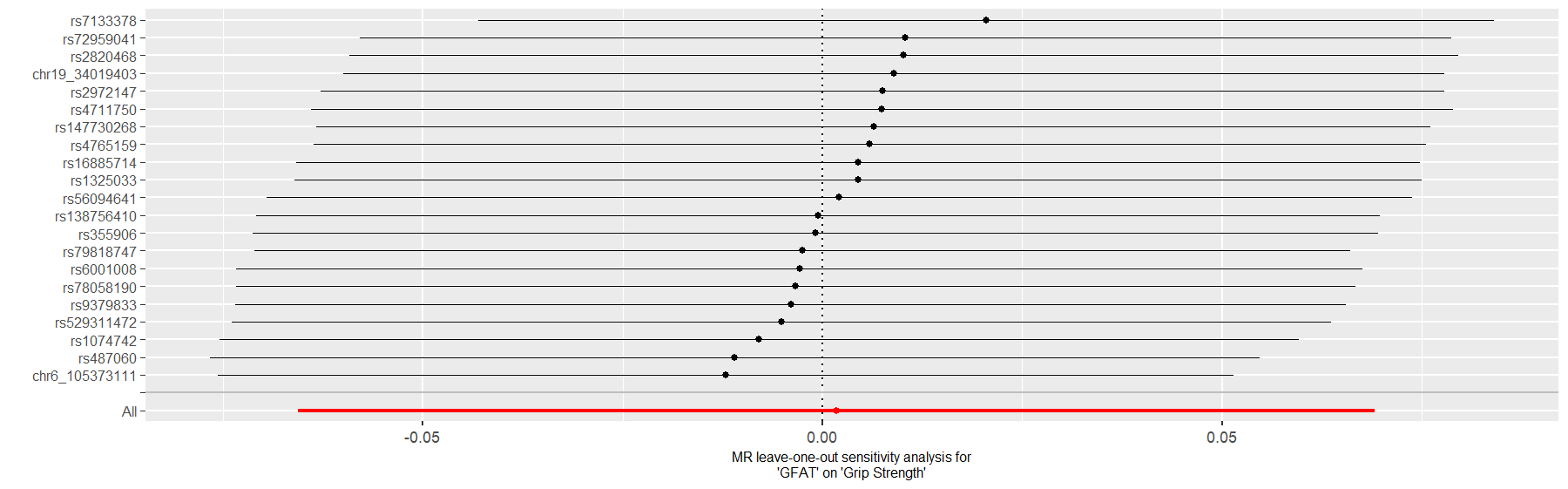


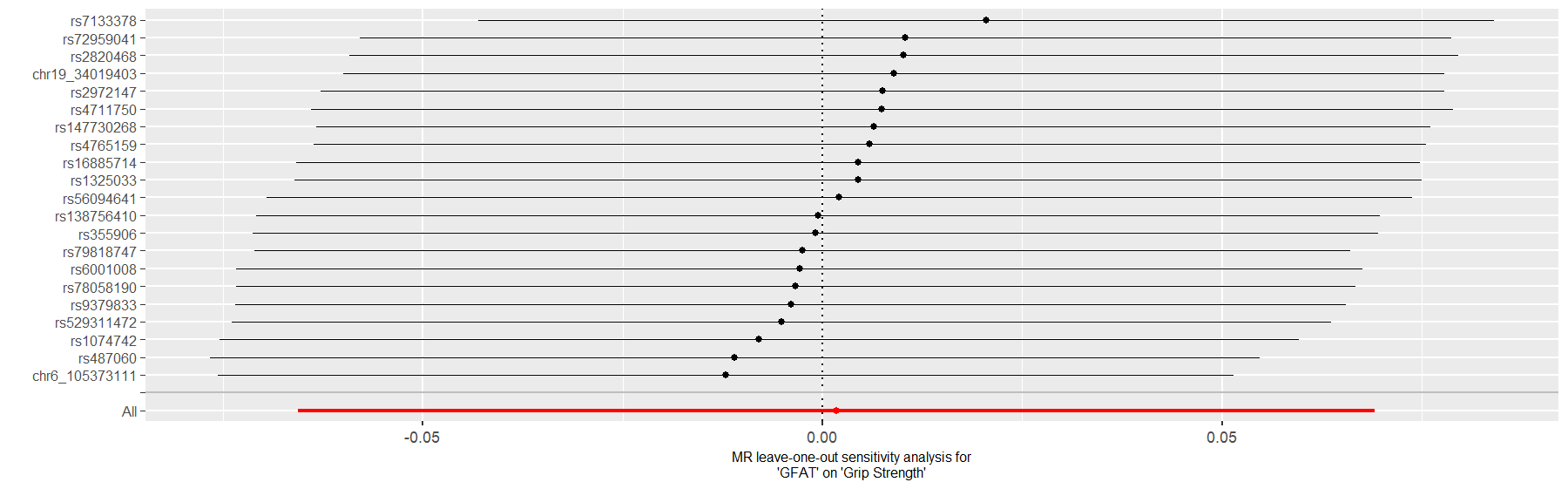
Males


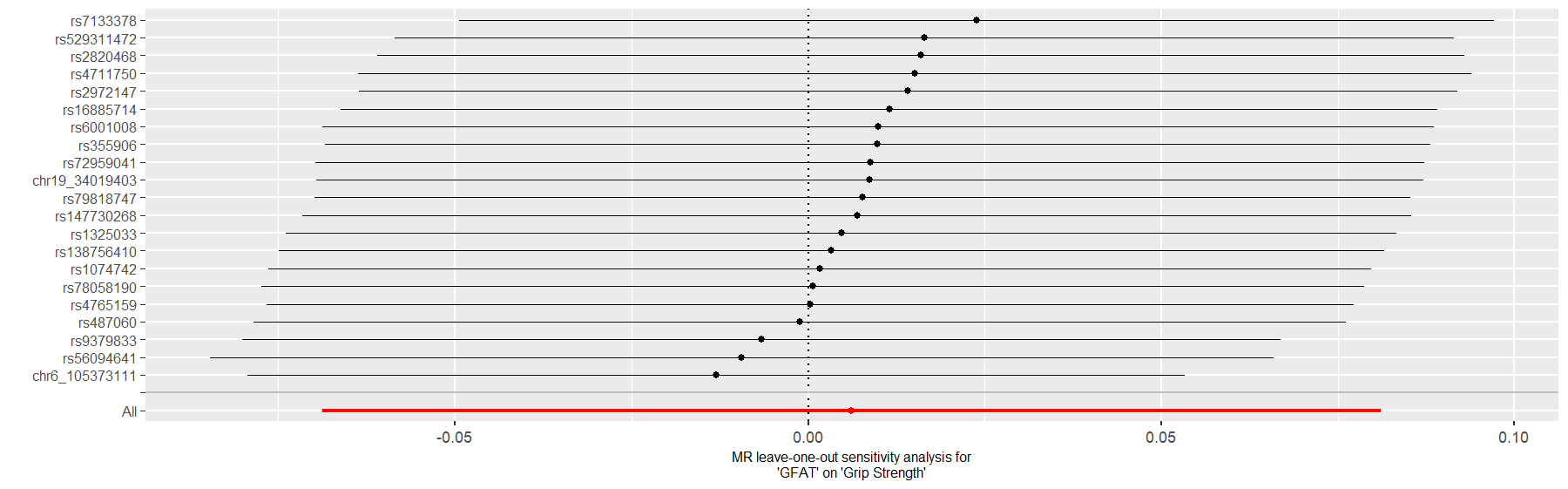
Females

## Figure S15. Leave one SNP out analyses for MR of ATMFI on Grip Strength

Both


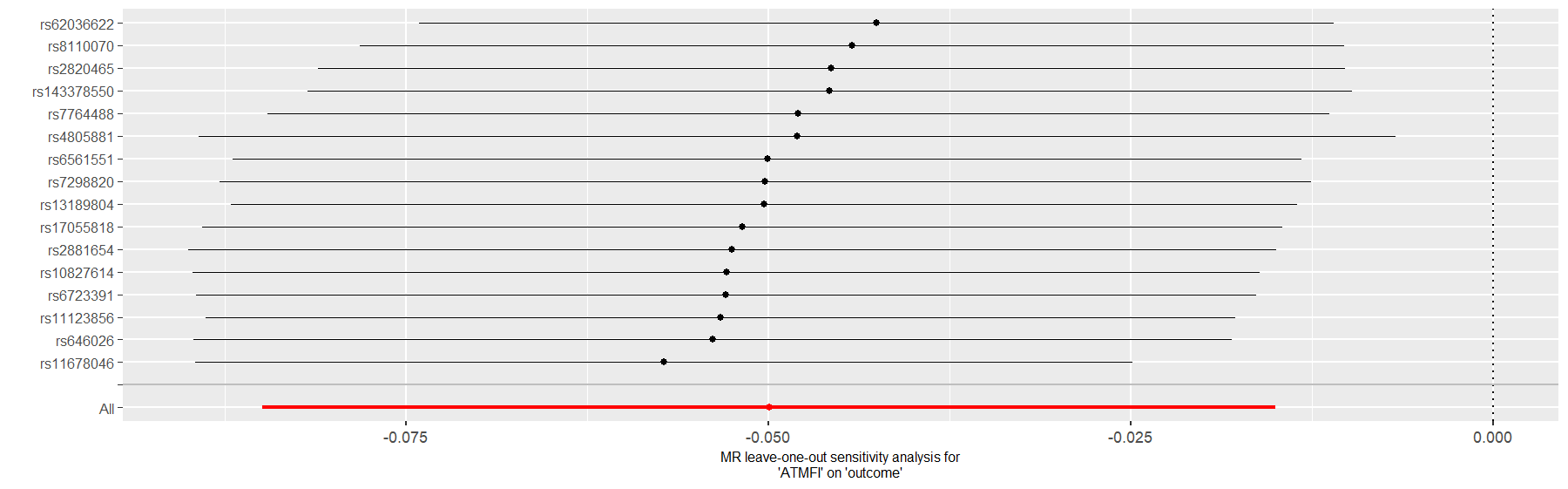


Males


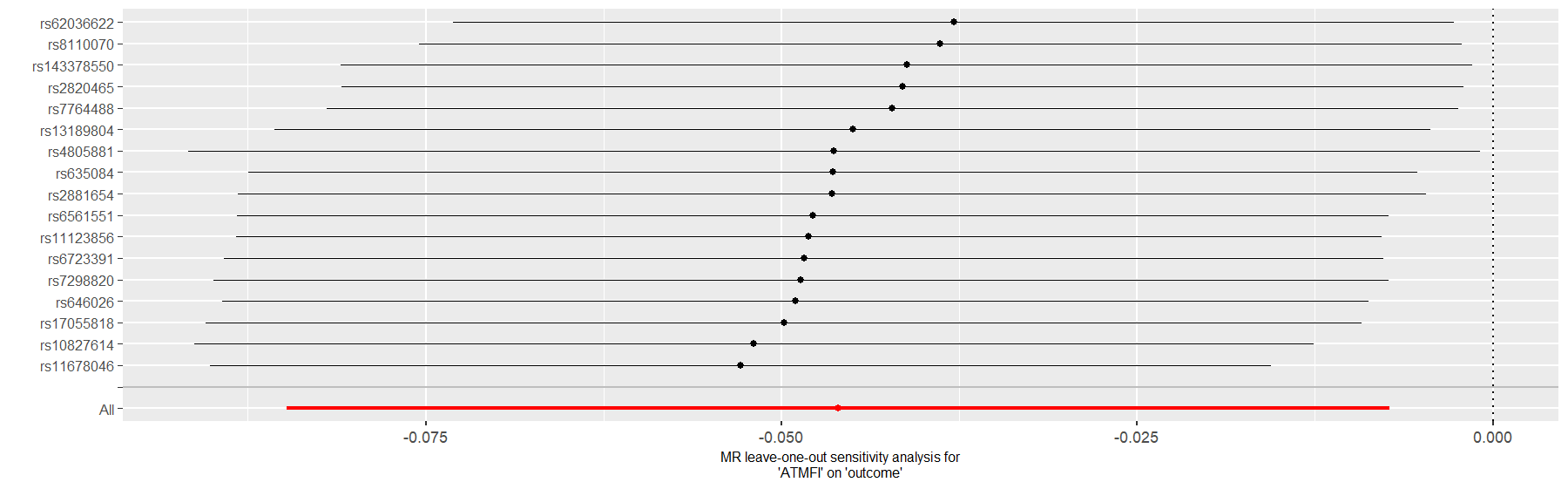


Females


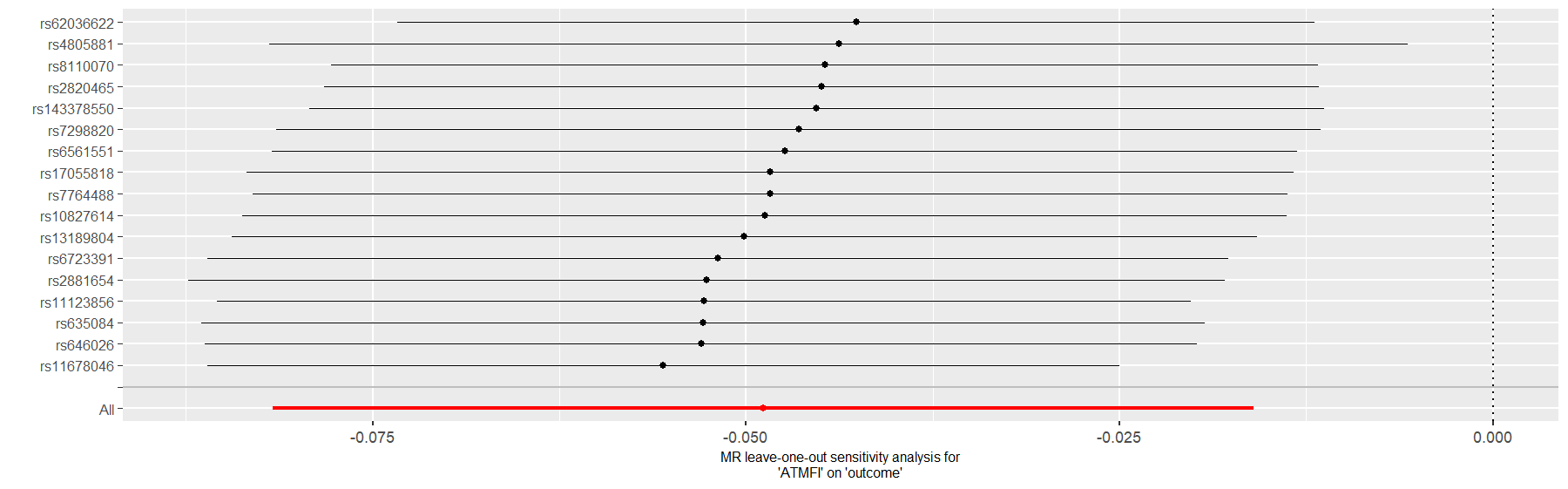


## Figure S16. Leave one SNP out analyses for MR of PTMFI on Grip Strength

Both


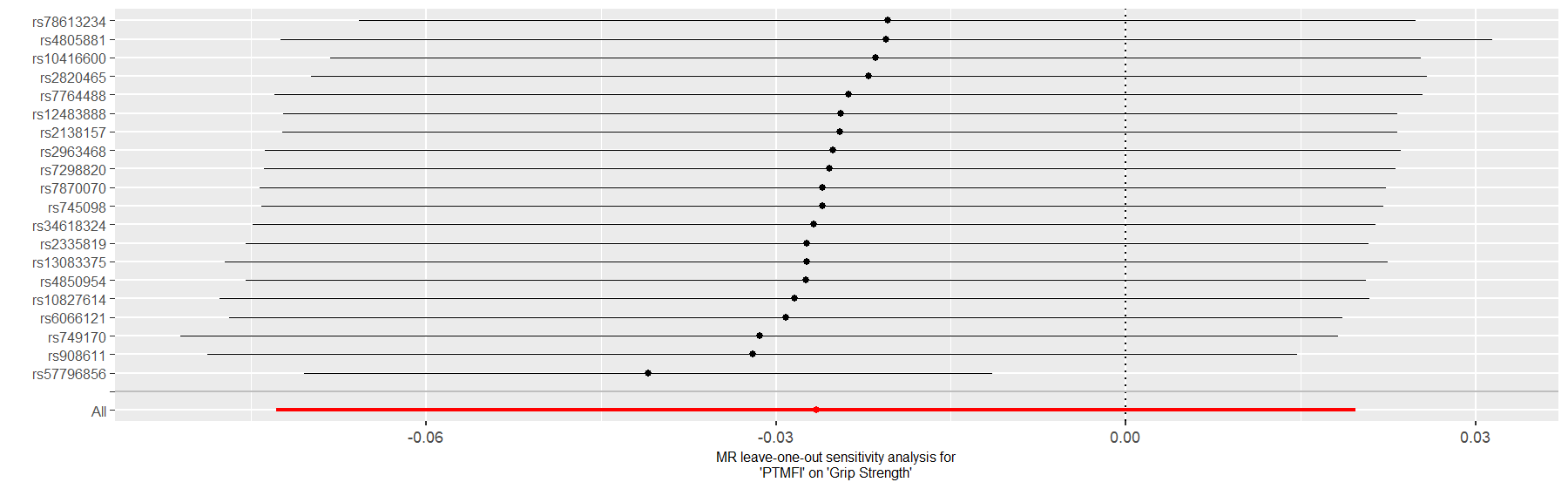


Males


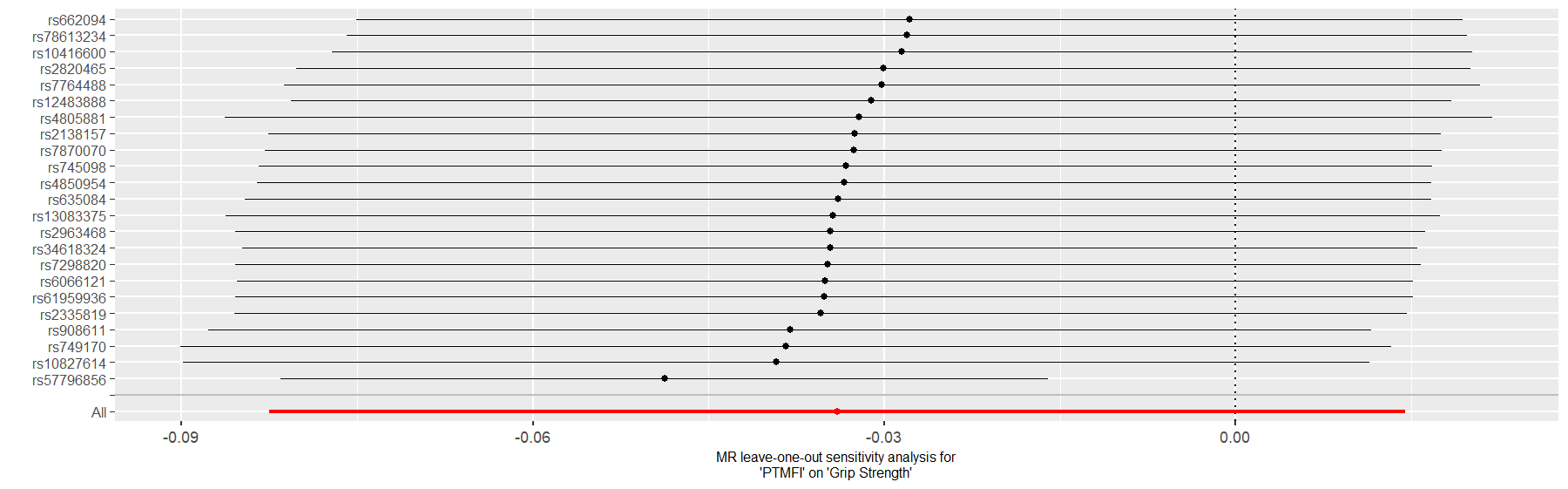


Females


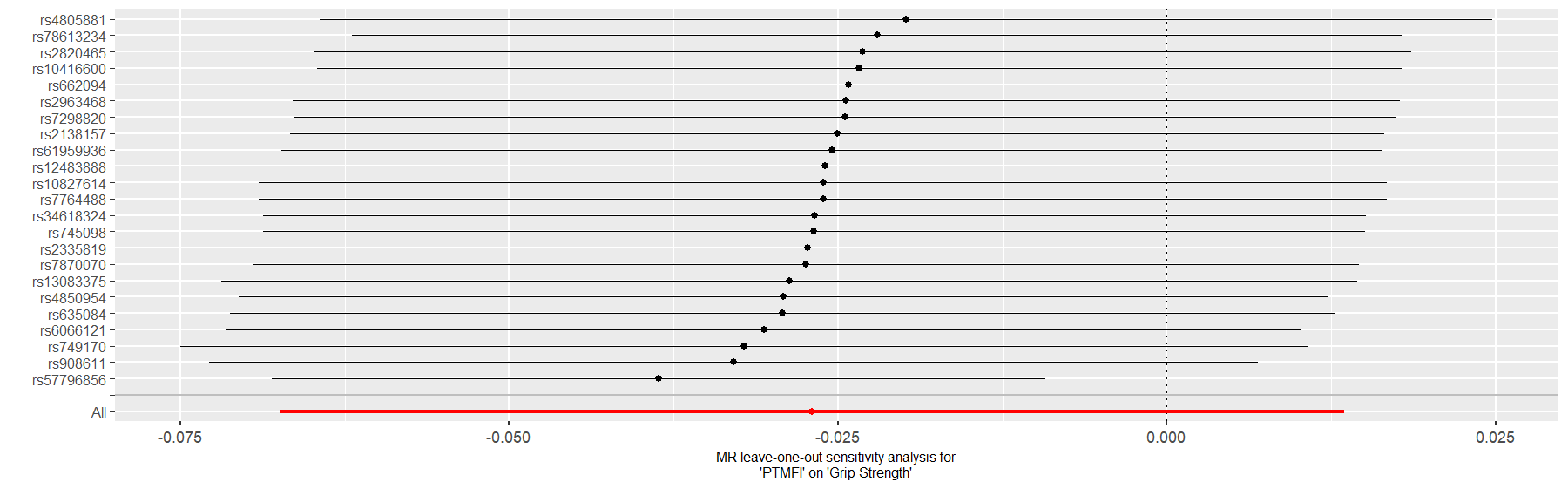


## Figure S17. Leave one SNP out analyses for MR of metabolically favourable adiposity on Grip Strength

Both


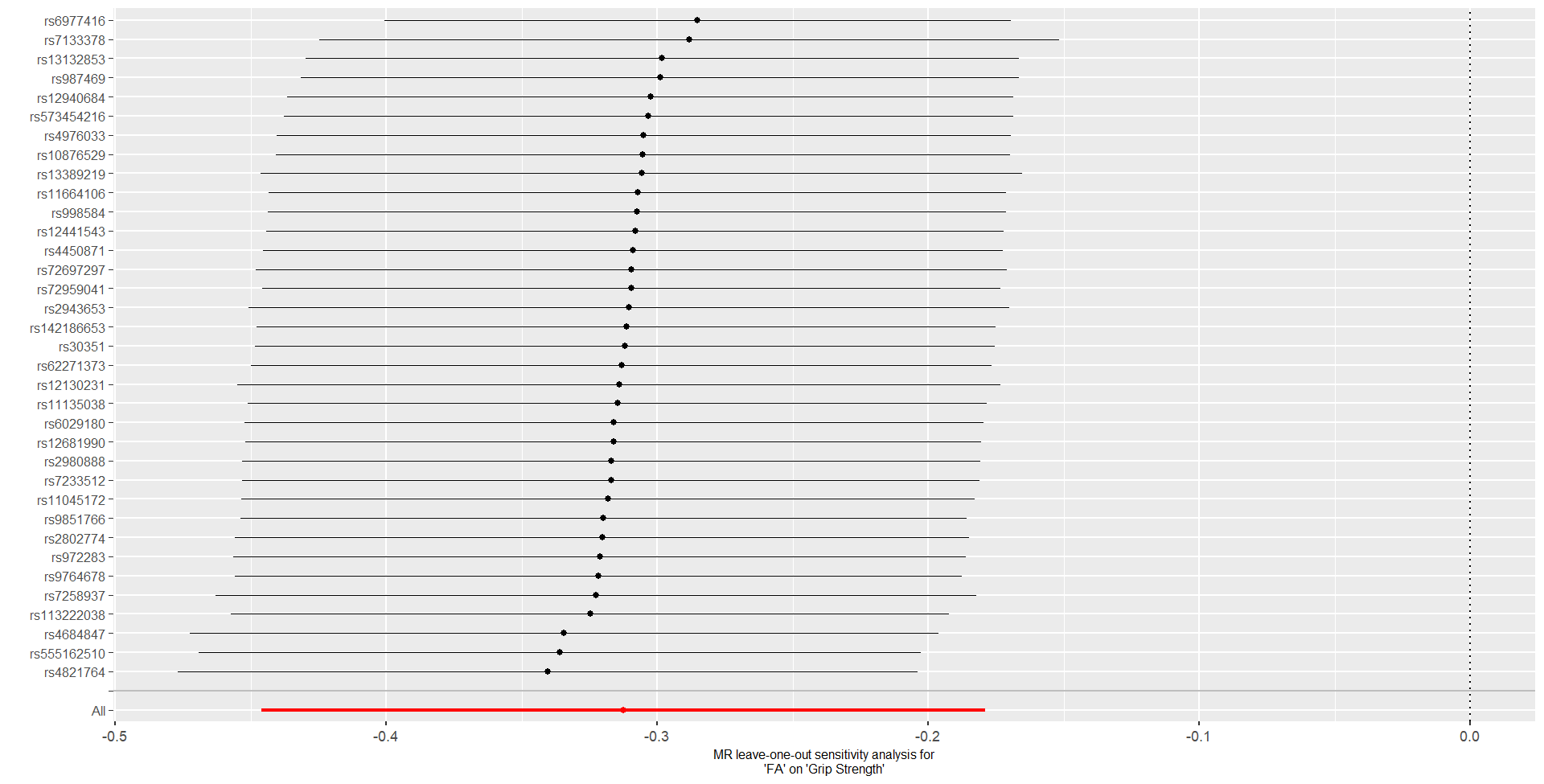


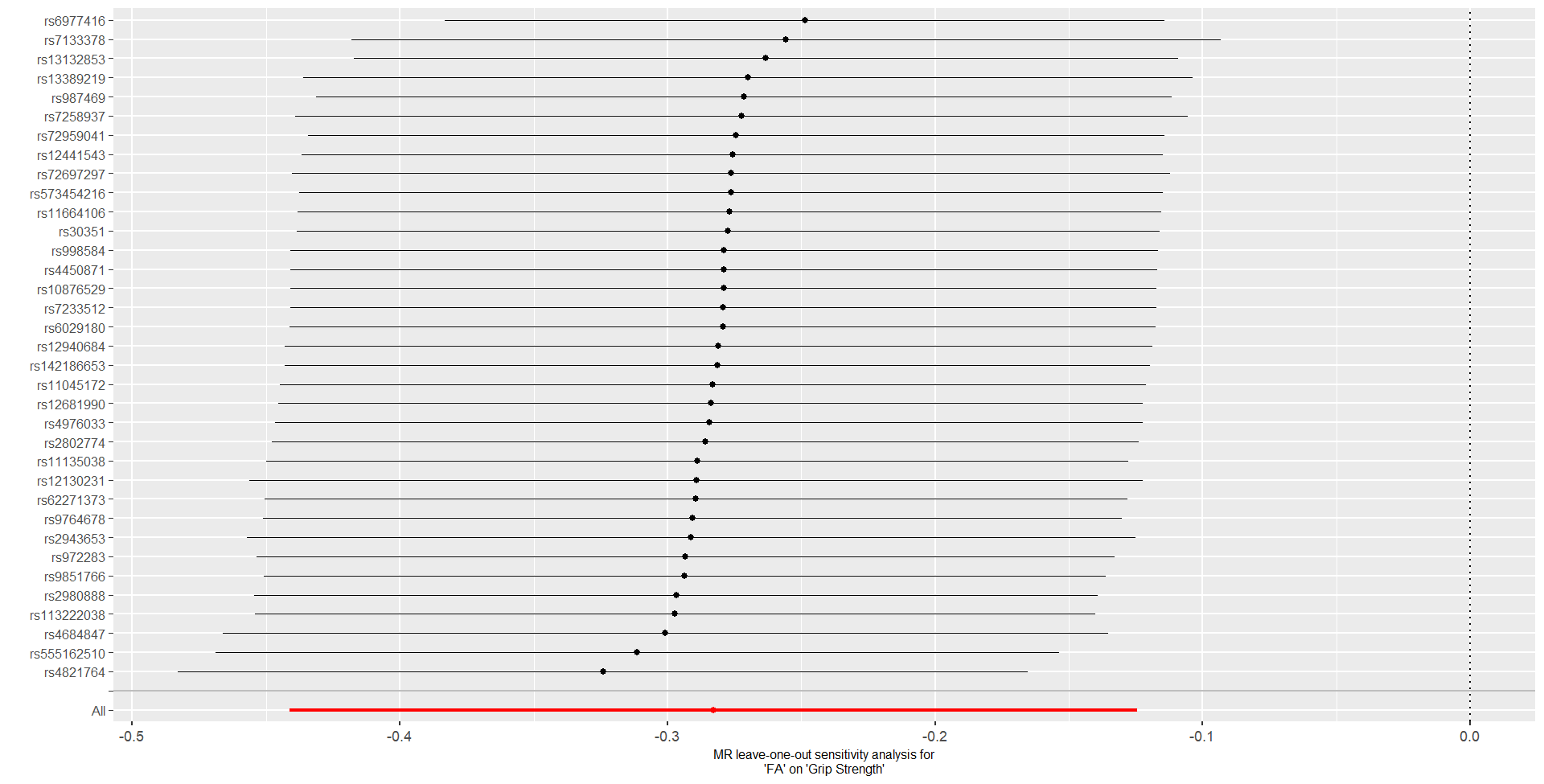
Males

Females


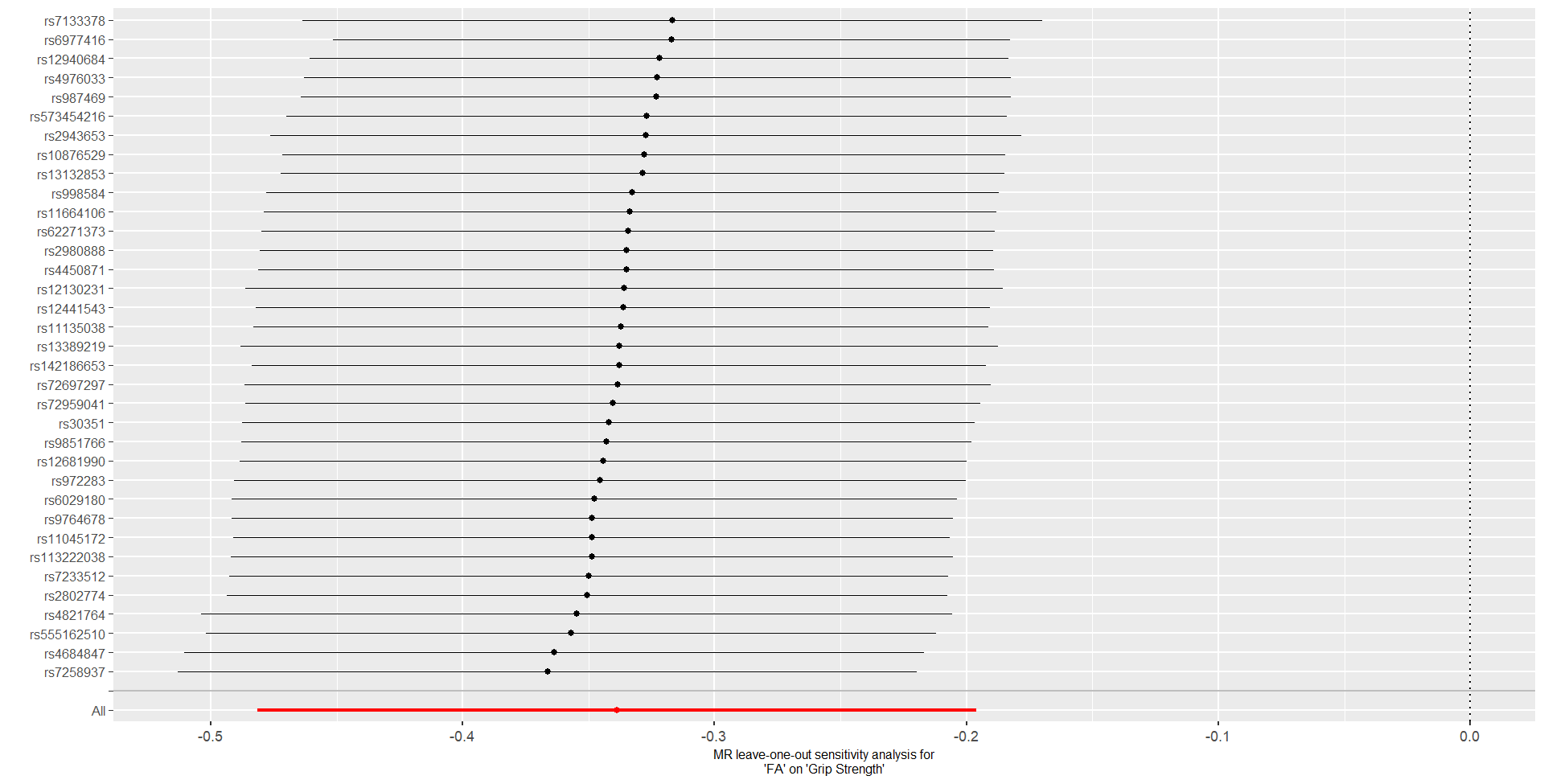


## Figure S18. Leave one SNP out analyses for MR of metabolically unfavourable adiposity on Grip Strength

Both


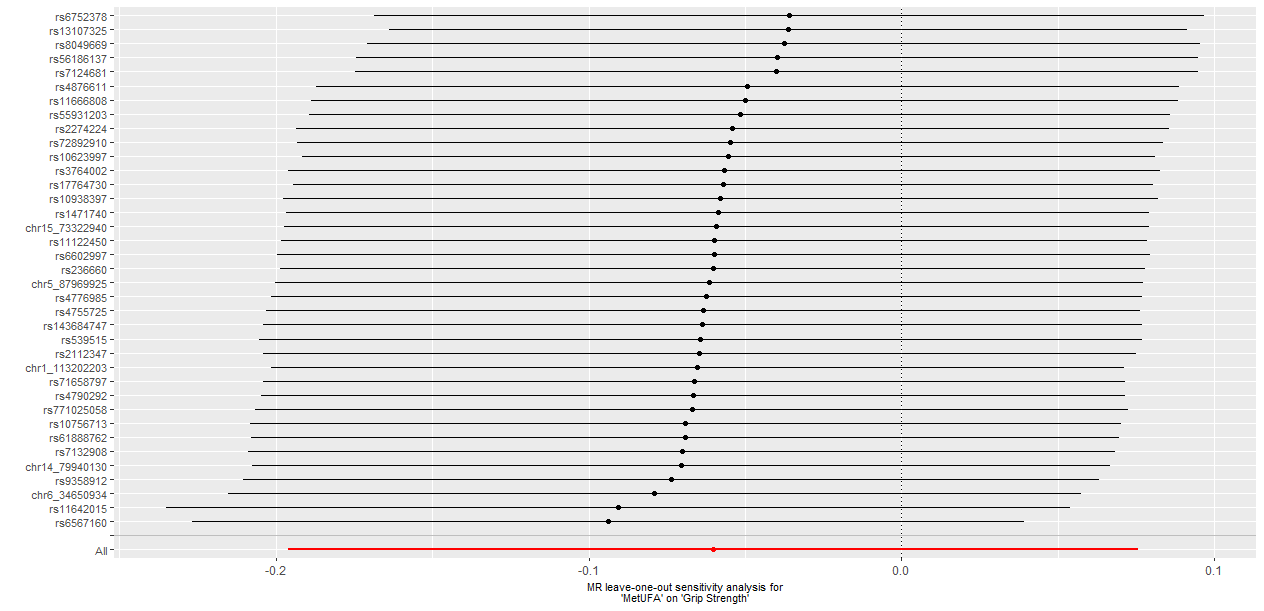


Males


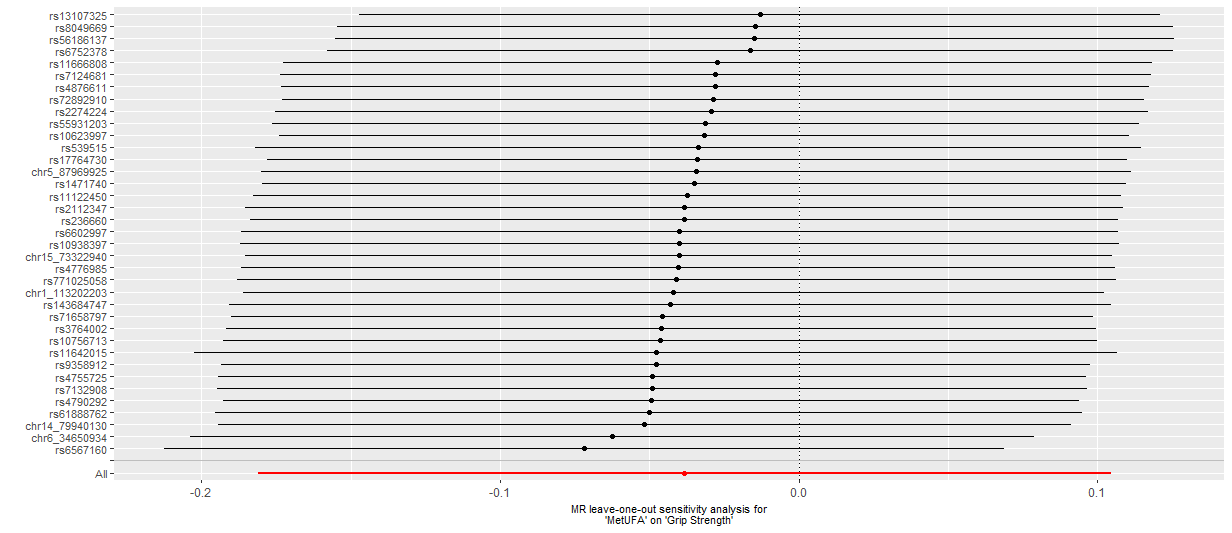


Females


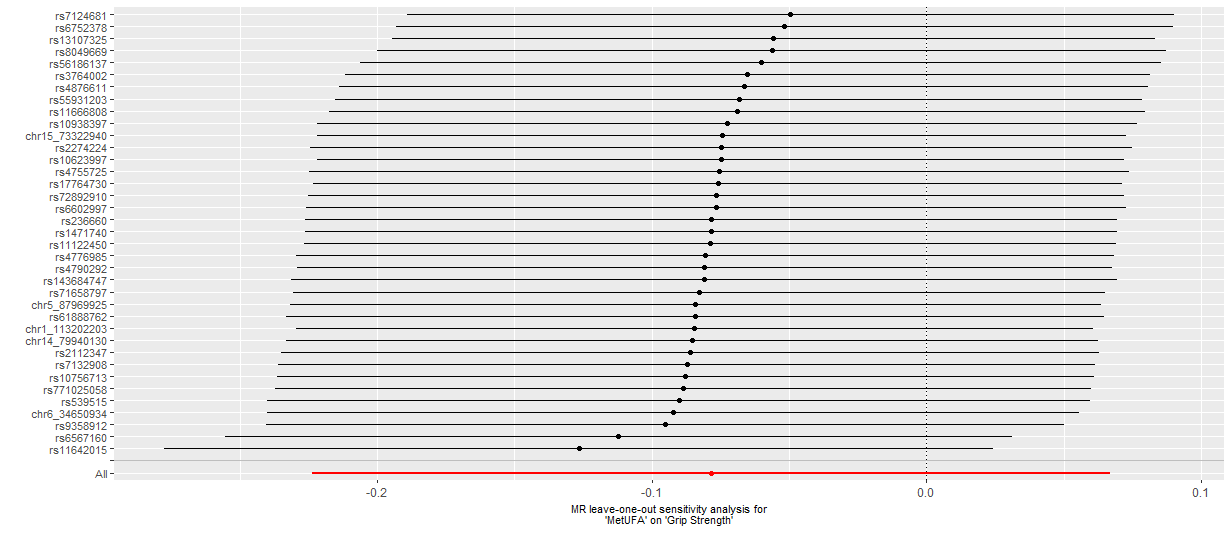


## Figure S19. Associations between adiposity measures and genetic scores with sex

**
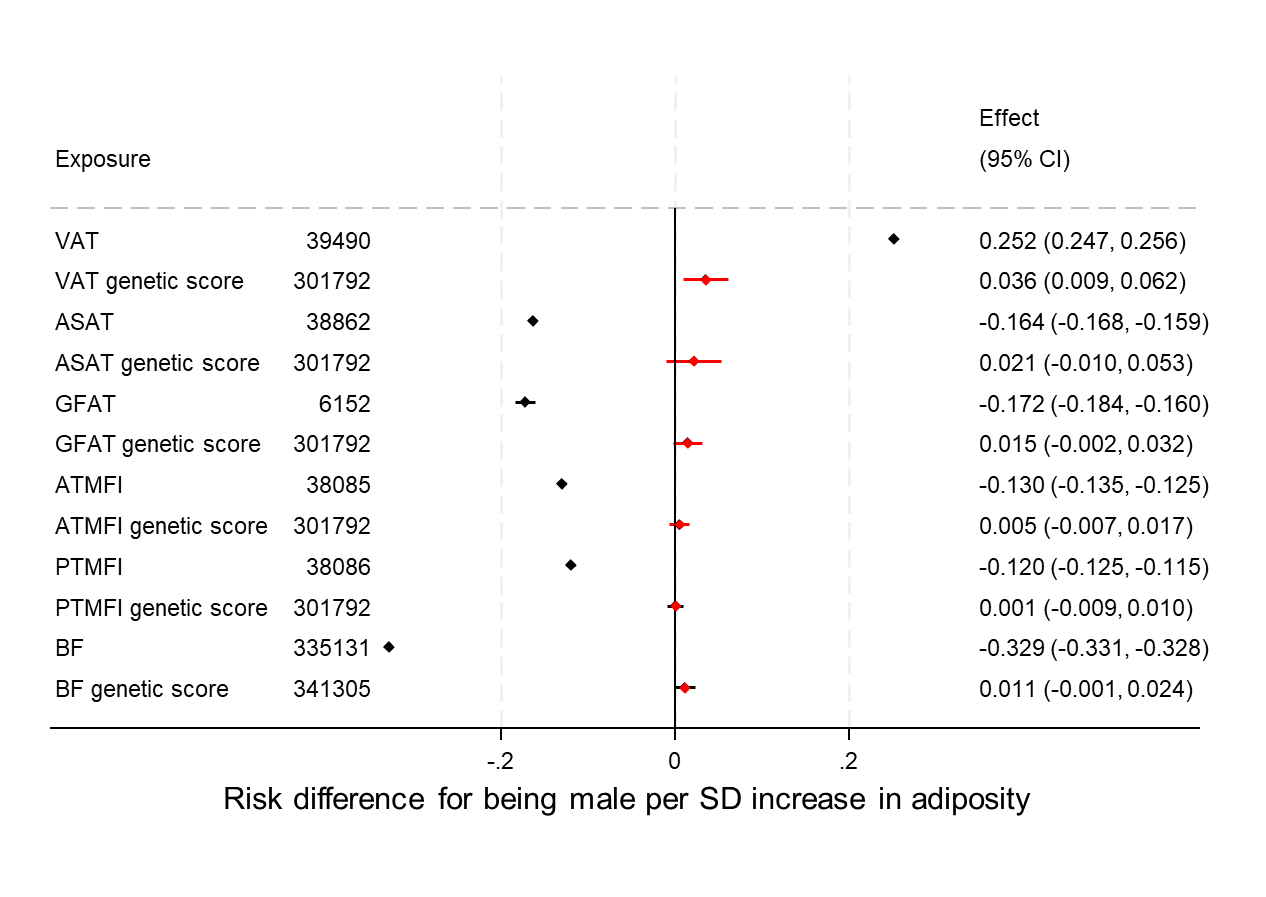
**

Associations are from linear regression on the risk difference scale within UK Biobank. Estimates in black represent phenotypic measures, estimates in Red represent the genetic scores. Genetic scores are weighted genomewide significant PRS (see Supplementary methods for details). Genetic scores have been scaled so that a unit increase is equivalent to an SD increase in adiposity measure. VAT: Visceral Adipose Tissue, ASAT: Abdominal Subcutaneous Adipose Tissue, GFAT: Gluteofemoral Adipose Tissue, ATMFI: Anterior Thigh Muscle Fat Infiltration, PTMFI: Posterior Thigh Muscle Fat Infiltration, BF: Body fat.

## Figure S20. Associations between adiposity measures and genetic scores with place of birth, North coordinate

**
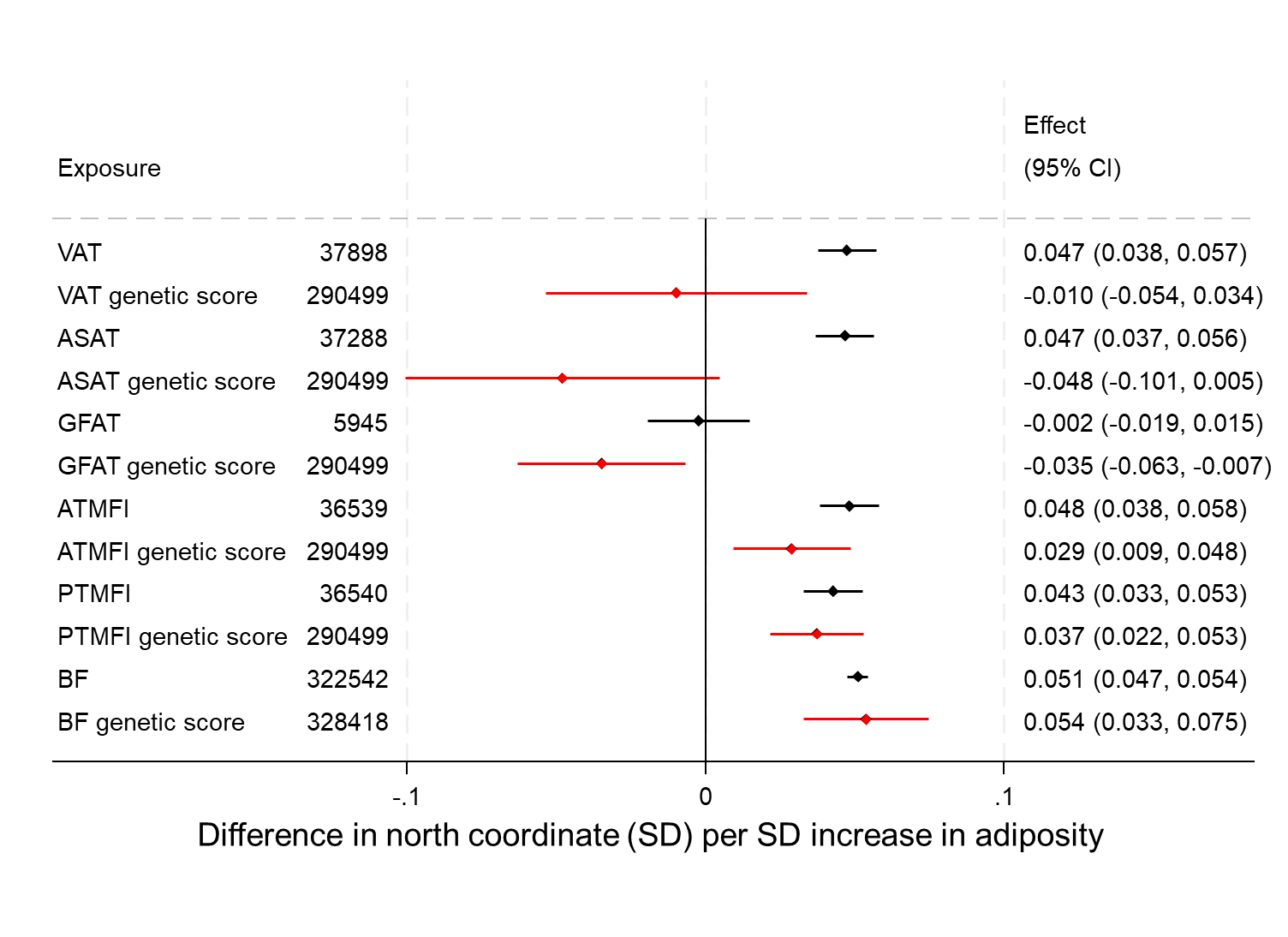
**

Associations are from linear regression within UK Biobank. Estimates in black represent phenotypic measures, estimates in Red represent the genetic scores. Genetic scores are weighted genomewide significant PRS (see Supplementary methods for details).Genetic scores have been scaled so that a unit increase is equivalent to an SD increase in adiposity measure. VAT: Visceral Adipose Tissue, ASAT: Abdominal Subcutaneous Adipose Tissue, GFAT: Gluteofemoral Adipose Tissue, ATMFI: Anterior Thigh Muscle Fat Infiltration, PTMFI: Posterior Thigh Muscle Fat Infiltration, BF: Body fat.

## Figure S21. Associations between adiposity measures and genetic scores with place of birth, East coordinate

**
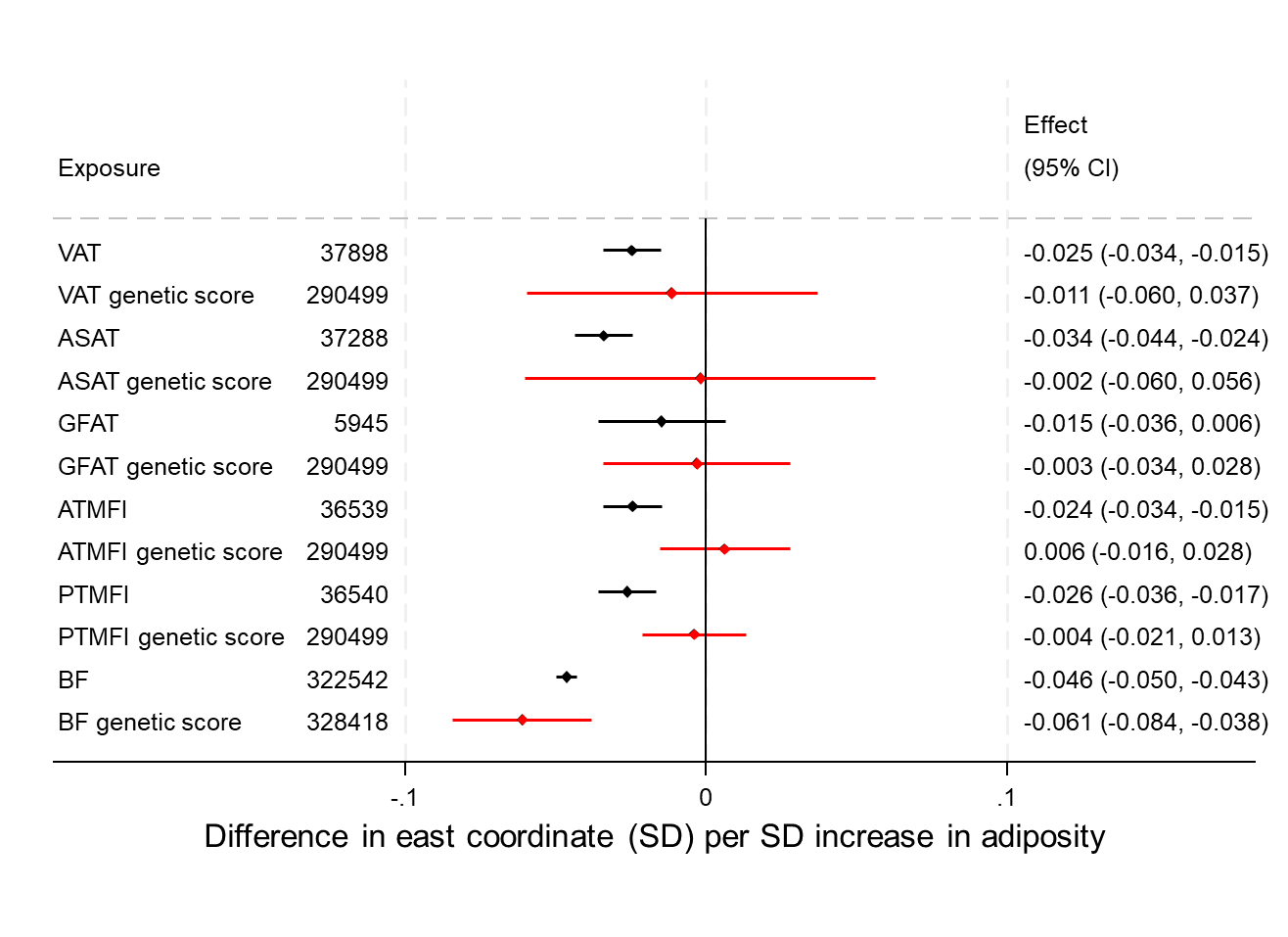
**

Associations are from linear regression within UK Biobank. Estimates in black represent phenotypic measures, estimates in Red represent the genetic scores. Genetic scores are weighted genomewide significant PRS (see Supplementary methods for details). Genetic scores have been scaled so that a unit increase is equivalent to an SD increase in adiposity measure. VAT: Visceral Adipose Tissue, ASAT: Abdominal Subcutaneous Adipose Tissue, GFAT: Gluteofemoral Adipose Tissue, ATMFI: Anterior Thigh Muscle Fat Infiltration, PTMFI: Posterior Thigh Muscle Fat Infiltration, BF: Body fat. Genetic scores are weighted genomewide significant scores.

# 3. Supplementary References

1. Linge J, Borga M, West J, Tuthill T, Miller MR, Dumitriu A, et al. Body Composition Profiling in the UK Biobank Imaging Study. Obesity (Silver Spring). 2018;26(11):1785-95.

2. Agrawal S, Klarqvist MDR, Diamant N, Stanley TL, Ellinor PT, Mehta NN, et al. BMI-adjusted adipose tissue volumes exhibit depot-specific and divergent associations with cardiometabolic diseases. Nat Commun. 2023;14(1):266.

3. van der Meer D, Gurholt TP, Sonderby IE, Shadrin AA, Hindley G, Rahman Z, et al. The link between liver fat and cardiometabolic diseases is highlighted by genome-wide association study of MRI-derived measures of body composition. Commun Biol. 2022;5(1):1271.

4. Machiela MJ, Chanock SJ. LDlink: a web-based application for exploring population-specific haplotype structure and linking correlated alleles of possible functional variants. Bioinformatics. 2015;31(21):3555-7.

5. Oscanoa J, Sivapalan L, Gadaleta E, Dayem Ullah AZ, Lemoine NR, Chelala C. SNPnexus: a web server for functional annotation of human genome sequence variation (2020 update). Nucleic Acids Res. 2020;48(W1):W185-W92.

6. Martin S, Tyrrell J, Thomas EL, Bown MJ, Wood AR, Beaumont RN, et al. Disease consequences of higher adiposity uncoupled from its adverse metabolic effects using Mendelian randomisation. Elife. 2022;11.

7. Agrawal S, Wang M, Klarqvist MDR, Smith K, Shin J, Dashti H, et al. Inherited basis of visceral, abdominal subcutaneous and gluteofemoral fat depots. Nat Commun. 2022;13(1):3771.

8. Bulik-Sullivan BK, Loh PR, Finucane HK, Ripke S, Yang J, Schizophrenia Working Group of the Psychiatric Genomics C, et al. LD Score regression distinguishes confounding from polygenicity in genome-wide association studies. Nat Genet. 2015;47(3):291-5.

9. Martin S, Cule M, Basty N, Tyrrell J, Beaumont RN, Wood AR, et al. Genetic Evidence for Different Adiposity Phenotypes and Their Opposing Influences on Ectopic Fat and Risk of Cardiometabolic Disease. Diabetes. 2021;70(8):1843-56.

10. Mbatchou J, Barnard L, Backman J, Marcketta A, Kosmicki JA, Ziyatdinov A, et al. Computationally efficient whole-genome regression for quantitative and binary traits. Nat Genet. 2021;53(7):1097-103.

11. Bowden J, Spiller W, Del Greco MF, Sheehan N, Thompson J, Minelli C, Davey Smith G. Improving the visualization, interpretation and analysis of two-sample summary data Mendelian randomization via the Radial plot and Radial regression. Int J Epidemiol. 2018;47(4):1264-78.

12. Burgess S, Davies NM, Thompson SG. Bias due to participant overlap in two-sample Mendelian randomization. Genet Epidemiol. 2016;40(7):597-608.

13. Sanderson E, Spiller W, Bowden J. Testing and correcting for weak and pleiotropic instruments in two-sample multivariable Mendelian randomization. Stat Med. 2021;40(25):5434-52.

14. Sanderson E, Richardson TG, Hemani G, Davey Smith G. The use of negative control outcomes in Mendelian randomization to detect potential population stratification. Int J Epidemiol. 2021;50(4):1350-61.

15. UK Biobank: deriving the grid coordinates. March 2024. Available at: <https://biobank.ndph.ox.ac.uk/showcase/ukb/docs/UKgrid.pdf>.

16. Davies NM, Thomas KH, Taylor AE, Taylor GMJ, Martin RM, Munafo MR, Windmeijer F. How to compare instrumental variable and conventional regression analyses using negative controls and bias plots. Int J Epidemiol. 2017;46(6):2067-77.
